# Systematic Literature Review of the Epidemiological Characteristics of Pneumococcal Disease Caused by the Additional Serotypes Covered by the 20-Valent Pneumococcal Conjugate Vaccine

**DOI:** 10.3390/microorganisms11071816

**Published:** 2023-07-15

**Authors:** Estelle Méroc, Mark A. Fletcher, Germaine Hanquet, Mary P. E. Slack, Marc Baay, Kyla Hayford, Bradford D. Gessner, Lindsay R. Grant

**Affiliations:** 1 P95 Epidemiology & Pharmacovigilance, Koning Leopold III-laan 1, 3001 Leuven, Belgium; estelle.meroc@p-95.com (E.M.); germaine.hanquet@p-95.com (G.H.); marc.baay@p-95.com (M.B.); 2 Emerging Markets Medical Affairs, Vaccines, Pfizer, 23–25 Av. du Dr Lannelongue, 75014 Paris, France; mark.a.fletcher@pfizer.com; 3 School of Medicine & Dentistry, Griffith University Gold Coast Campus, Parklands Drive, Southport, QLD 4222, Australia; mary.slack@pfizer.com; 4 Medical Development and Scientific Clinical Affairs, Pfizer Vaccines, 500 Arcola Road, Collegeville, PA 19426, USA; kyla.hayford@pfizer.com (K.H.); bradford.gessner@pfizer.com (B.D.G.)

**Keywords:** 20-valent pneumococcal conjugate vaccine, invasive pneumococcal disease, proportion, incidence, severity, antimicrobial non-susceptibility, PCV20, PCV15, *Streptococcus pneumoniae*

## Abstract

Higher valency pneumococcal conjugate vaccines (PCV15 and PCV20) have been developed to address the disease burden of current non-vaccine serotypes. This review describes the epidemiological characteristics of serotypes beyond PCV13 (serotypes 8, 10A, 11A, 12F, 15B/C, 22F, and 33F; PCV20nonPCV13 serotypes). Peer-reviewed studies published between 1 January 2010 (the year PCV13 became available) and 18 August 2020 were systematically reviewed (PROSPERO number: CRD42021212875). Data describing serotype-specific outcomes on disease proportions, incidence, severity, and antimicrobial non-susceptibility were summarized for individual and aggregate PCV20nonPCV13 serotypes by age group and by type and duration of pediatric PCV immunization program. Of 1168 studies, 127 (11%) were included in the analysis. PCV20nonPCV13 serotypes accounted for 28% of invasive pneumococcal disease (IPD), although the most frequent serotypes differed between children (10A, 15B/C) and adults (8, 12F, 22F). In children, serotype 15B/C tended to be more frequently associated with pneumococcal meningitis and acute otitis media; in adults, serotype 8 was more frequently associated with pneumonia and serotype 12F with meningitis. Serotypes 10A and 15B/C in children and 11A and 15B/C in adults were often associated with severe IPD. Serotype 15B/C was also among the most frequently identified penicillin/macrolide non-susceptible PCV20nonPCV13 serotypes. These results could inform decision making about higher valency PCV choice and use.

## 1. Introduction

The prevention of pneumococcal disease in children and adults has long been a public health priority. The potential to achieve this goal was substantially enhanced by the introduction of pneumococcal conjugate vaccines (PCVs). Unlike plain polysaccharide vaccines, PCVs prevent disease across the age spectrum, including young infants and the oldest adults [1]. PCVs induce a T cell-mediated immune response that leads to prolonged memory and boosting following subsequent exposure; they reduce carriage and thus provide indirect protection to unvaccinated persons; and they have documented efficacy against vaccine-type mucosal disease such as non-bacteremic pneumonia in adults [2] and otitis media in children [3]. The first licensed PCV contained seven serotypes (4, 6B, 9V, 14, 18C, 19F, and 23F) and was followed by higher order formulations: PCV10 (containing PCV7 serotypes plus 1, 5, and 7F) and PCV13 (containing PCV10 serotypes plus 3, 6A, and 19A). As of December 2020, PCVs had been included in the pediatric national immunization programs (NIPs) of 160 countries globally, with PCV13 used in more than 130 pediatric NIPs [4]. For adults, countries have issued recommendations for risk-based or age-based pneumococcal vaccination (many providing PCV13 sequentially with 23-valent pneumococcal polysaccharide vaccine [PPSV23]) [5], although few have also included public funding for reimbursement of vaccine costs.

Despite the successes of pediatric and, in some settings, adult national PCV programs, pneumococcal disease due to non-vaccine serotypes (NVTs) persists. PCVs reduce acquisition of vaccine serotypes (VTs), creating a new niche for NVTs to occupy leading to an increase in NVT circulation. Whether serotype replacement in the nasopharynx leads to increased NVT disease incidence depends on several factors, including the invasiveness potential of each NVT, antibiotic non-susceptibility of each NVT and antimicrobial use in the community, and host factors such as age and underlying medical conditions [6,7,8]. To address this issue, two new vaccines have been licensed in adults: PCV15 (PCV13 serotypes plus 22F and 33F) and PCV20 (PCV15 serotypes plus 8, 10A, 11A, 12F, and 15B). Each vaccine was licensed for adults based on comparative safety and immunogenicity to PCV13 [4,6]. PCV15 and PCV20 have been licensed, each for pediatric and adult use. The utility of these higher-valency vaccines will depend on the epidemiologic characteristics of pneumococcal disease due to these new serotypes, including disease proportion, disease incidence, disease severity, and propensity for antibiotic non-susceptibility in clinical isolates. Several multicenter studies and literature reviews have included some or all these additional serotypes, but the analyses were limited to the serotype distribution of IPD cases [9,10].

The objective of this current review was to describe the epidemiologic characteristics of the serotypes in higher-valency PCVs. Results are reported for the seven additional serotypes in PCV20 that are not in PCV13 (PCV20nonPCV13), specifically serotypes 8, 10A, 11A, 12F, 15B/C, 22F, and 33F, and for the five additional serotypes in PCV20 that are not in PCV15 (PCV20nonPCV15), specifically serotypes 8, 10A, 11A, 12F, and 15B/C.

## 2. Methods

The study protocol was registered on PROSPERO (international prospective register of systematic reviews) with registration number CRD42021212875, and the manuscript was prepared according to the critical domains of the AMSTAR2 guidelines.

### 2.1. Systematic Literature Review

The database search was conducted in Medline (via PubMed) and Embase to identify peer-reviewed studies published between 1 January 2010 (year when PCV13 became available) and 18 August 2020. The details of the search strategy with key words are provided in Text S1. Studies were eligible for inclusion if they: (1) described pneumococcal disease cases (i.e., excluding pneumococcal carriage data) including invasive pneumococcal disease (IPD) or Non-IPD, based on laboratory-confirmed diagnosis (established by microbiological culture, molecular detection assay, or antigen-based test) caused by any of the PCV20nonPCV13 serotypes; (2) reported at least one of the following serotype-specific outcomes: proportion, incidence rate (IR), severity data (e.g., case fatality ratio (CFR), mortality rate, intensive care unit admission rate, etc.), or antimicrobial non-susceptibility (restricting to penicillin and macrolide non-susceptibility or multidrug resistant (MDR), defined as resistance to at least one antibiotic from three or more different classes of antimicrobials according to the CDC definition: https://www.cdc.gov/narms/resources/glossary.html, accessed on 28 November 2022; (3) were conducted after 1 January 2010, the year when PCV13 became available; (4) contained primary data; and (5) were published in Dutch, English, French, German, Italian Portuguese, or Spanish. All types of study designs were eligible for inclusion; however, review publications were excluded but reference lists were screened to identify additional publications.

Titles and abstracts from the list of references were screened independently by two reviewers to identify studies that fulfilled the inclusion criteria. The full texts of all selected studies were then assessed for eligibility by the reviewers. The systematic literature review (SLR) was conducted in accordance with the guidelines from “Strengthening the Reporting of Observational Studies in Epidemiology” (STROBE) [11] and the standards of “Preferred Reporting Items for Systematic Reviews and Meta-Analyses” (PRISMA) [12].

### 2.2. Data Collection and Analysis

Data from eligible full-text articles were subsequently extracted by three reviewers each using a standardized form. The following serotype-specific outcome measures were collected for the PCV20nonPCV13 serotypes: specific number and total cases for each clinical presentation of pneumococcal disease to calculate serotype-specific and aggregate PCV20nonPCV13 proportions, IR, CFR, and proportion of isolates within a serotype that were antimicrobial non-susceptible or MDR.

Outcomes were stratified by current PCV (PCV10 or PCV13) in the pediatric NIP with duration of use or by no PCV in the NIP. Duration was defined as the number of years between the date of introduction of PCV10 or PCV13 in the NIP and the mid-year study period, irrespective of an initial PCV7 NIP (Table S1). For example, if a study reported data over a range of years (e.g., January 2014–December 2016), then the mid-year study period (e.g., January 2015) was used in the calculation of PCV use duration in the NIP (e.g., January 2015 minus NIP introduction date). “Initial period” was defined as a PCV NIP duration of <3 years, and “later period” was defined as ≥3 years duration of a PCV NIP. Outcome measures were also stratified by age group (children: <18 years or <5 years; adults: ≥18 years unless otherwise specified) and by clinical presentation as follows:

- Sterile site: inclusive of IPD defined as pneumococcus identified from any normally sterile site (blood, cerebrospinal fluid, pleural fluid, etc.) [13]. IPD clinical presentations included bacteremia/sepsis, meningitis, or pneumonia. “Other IPD” was defined as infrequently occurring infections of other normally sterile sites (joint fluid, pericardial fluid, peritoneal fluid, and infected bone, among others).
- Non-sterile site: inclusive of Non-IPD defined as pneumococcus identified from a site that is not considered to be normally sterile. Non-IPD clinical presentations included bronchitis and non-bacteremic pneumonia (respiratory tract sample, identified by sputum), acute otitis media (AOM, identified from middle ear fluid), or sinusitis (sinuses swab).
- Undifferentiated site: inclusive of pneumococcal disease that could not be differentiated in the reported results, whether by site (sterile or non-sterile) or by clinical presentation (invasive or non-invasive disease). Most often, undifferentiated diseases included pneumococcal pneumonia reported together that was bacteremic (IPD) or non-bacteremic (Non-IPD).

An ‘IPD, presentation unspecified’ or ‘Non-IPD, presentation unspecified’ category refers to cases where the type of clinical presentation was reported (i.e., IPD or Non-IPD) but not the precise clinical presentation (i.e., presentation unspecified).

The PCV20nonPCV13 serotypes were defined as serotypes 8, 10A, 11A, 12F, 15B/C, 22F, and 33F. Because of structural similarity between serotypes 15B and 15C [7,14] and the possibility of cross-reactivity, 15C also was defined as a PCV20nonPCV13 serotype. PCV20nonPCV15 serotypes were defined as serotypes 8, 10A, 11A, 12F, and 15B/C.

Serotype-specific proportions were calculated, in which the denominator was the total number of pneumococcal isolates that had been serotyped in the study. Calculation of the proportions of all PCV20nonPCV13 serotypes and all PCV20nonPCV15 was restricted to studies that reported cases for all of these seven and five serotypes, respectively.

Meta-analysis of the proportion of IPD cases due to an individual serotype was performed using a binomial, random effects, meta-analytic model for each PCV20nonPCV13 serotype independently. Few countries identified in this SLR had a PCV10 NIP or a no PCV NIP; consequently, only PCV13 NIPs were included in the meta-analyses. The statistical heterogeneity between studies was assessed by estimating the I2 statistic [8]. All statistical analyses were conducted with R version 4.0.2.

Lastly, a risk of bias tool, adapted from the Newcastle–Ottawa Scale [15], was used to assess the quality of the studies included in the SLR (Table S2). The scale establishes low (risk score: 0 out of 6), moderate (1 to 2 out of 6), or serious/critical risk (≥3 out of 6). On studies with low risk of bias, a sensitivity analysis of the meta-analysis was carried out excluding studies with moderate or serious/critical risk of bias.

## 3. Results

### 3.1. Studies Included in the Systematic Literature Review

We performed the database search on 18 August 2020, identifying 1168 unique references. Of these, 127 (11%) studies were included in the SLR (Figure S1). In total, 41 of the 127 studies contributed data to multiple analyses, reporting data from multiple World Health Organization (WHO) regions, from different age groups, or across multiple clinical presentations.

Of the 127 included studies, 26 were conducted in the Americas, 64 were conducted in Europe, and 24 were conducted in the Western Pacific, per WHO region definitions. Respectively, 112, 10, and 5 studies reported data from countries with a PCV13 NIP, a PCV10 NIP, or no PCV NIP. Thirty-six studies provided information for all PCV20nonPCV13 serotypes, and forty-two provided information for all PCV20nonPCV15 serotypes (Table 1). The 127 studies reported data on children (86 studies), adults (56 studies), or all ages together indiscriminately (13 studies); by outcome, most studies described IPD (sterile site) (94 studies), followed by Non-IPD (non-sterile site) (27 studies), and all pneumococcal disease (undifferentiated) (19 studies). The breakdown of selected studies, by WHO region, age group, and clinical presentation type, is shown in Figure S2. Further study characteristics are described in Table S3.

Table 1 summarizes the pooled proportion of cases by clinical presentation of pneumococcal disease, due to all PCV20nonPCV13 or all PCV20nonPCV15 serotypes, based only on the studies that reported data for all seven PCV20nonPCV13 serotypes (n = 36 studies) or all five PCV20nonPCV15 serotypes (n = 42 studies). Among all age groups, the PCV20nonPCV13 and the PCV20nonPCV15 serotypes accounted for close to one-third and one-fifth of IPD cases, respectively. Among children, the PCV20nonPCV13 and PCV20nonPCV15 serotypes accounted for, respectively, 27.8% (n = 17 studies) and 19.7% (n = 19 studies) of overall IPD. Among adults, these two proportions represented 27.5% (n = 16 studies) and 19.7% (n = 18 studies), respectively.

### 3.2. Proportion of PCV20nonPCV13 Serotypes Causing IPD

**Figure 1:**
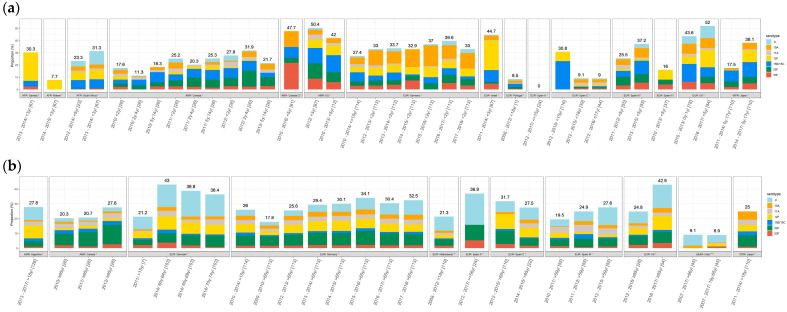
PCV20nonPCV13 serotype proportion of IPD isolates by country for 25 studies that reported data for all seven PCV20nonPCV13 serotypes: (**a**) Children (**b**) Adults. * PCV13 NIP. ** PCV10 NIP. *** No PCV NIP. Only four cases were identified among children from Spain A. Abbreviations: AFR = African WHO region; AMR = Americas; EUR = Europe; SEAR = Southeast Asia; WPR = Western Pacific; Canada Q = Quebec; Spain A = Andalusia; Spain C = Catalonia; Spain M = Madrid; Spain N = Navarra. Reference numbers for each study are provided in the Supplementary Materials.

Figure 1 displays the distribution of the individual PCV20nonPCV13 serotypes in the 25 studies that reported IPD cases (‘IPD overall’ in Table 1) for all seven PCV20nonPCV13 serotypes. All countries included in this analysis used PCV13 in the NIP except the Netherlands which used PCV10. The PCV20nonPCV13 serotypes ranged from 0% to 52% of all IPD cases in children (panel 1a) and from 9% to 55% in adults (panel 1b). Studies from Canada, Germany, Spain, and Japan reported IPD cases stratified by year or groups of years, and in these studies, overall proportions for the PCV20nonPCV13 serotypes tended to increase over time in both children and adults (Figure 1). Among the PCV20nonPCV13 serotypes, the proportion of IPD due to serotype 10A (Germany) and 22F (Canada) increased among children, while serotype 8 (Germany, Spain—Madrid, United Kingdom (UK)) increased among adults.

### 3.3. Proportion of PCV20nonPCV13 Serotypes by Clinical Presentation and PCV13 Use Period

Pooled proportions of the individual PCV20nonPCV13 serotypes by clinical presentation, age group, and PCV NIP duration are in Table S4. Among children in the PCV13 NIP initial period, serotype 15B/C was frequently identified across the spectrum of clinical presentations. Among children during the PCV13 NIP later period, the frequency of ‘IPD, presentation unspecified’ was comparable for serotypes 10A, 12F, and 15B/C. Among frequent causes of other clinical presentations were serotype 11A (other IPD; pneumococcal AOM; all pneumococcal pneumonia), serotype 12F (pneumococcal bacteremia; other IPD), and serotype 15B/C (in all clinical presentations except pneumococcal pneumonia). Each PCV20nonPCV13 serotype was more frequent among adults in the PCV13 NIP later period than in the PCV13 NIP initial period. Among adults in the PCV13 NIP later period, serotypes 8 and 12F were most frequently detected (pneumococcal bacteremia, pneumococcal meningitis, bacteremic pneumococcal pneumonia, and other IPD; IPD presentation unspecified; all pneumococcal pneumonia). In addition to serotypes 8 and 12F, for ‘IPD, presentation unspecified’, serotype 22F was also among the most frequently detected.

### 3.4. Proportion of PCV20nonPCV13 Serotypes Causing IPD by PCV13 Use Period

**Figure 2:**
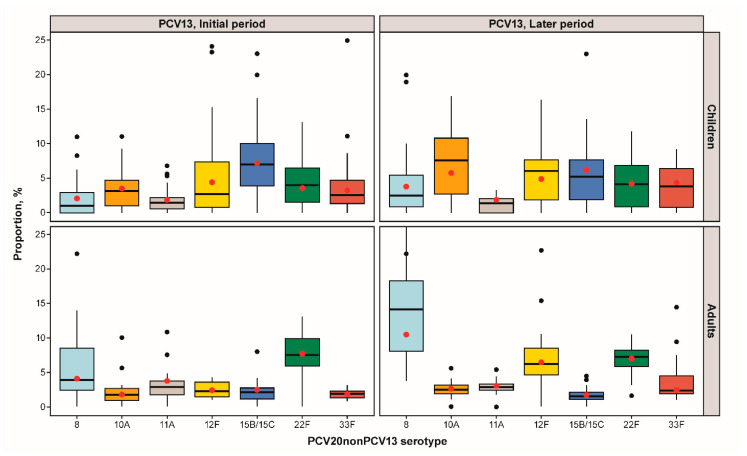
IPD PCV20nonPCV13 serotype proportions stratified by age group and pediatric PCV13 program duration: proportion distribution and meta-analysis point estimate (red dot).

Figure 2 and Table S5 show the pooled proportions for IPD cases in PCV13 NIP countries. Studies in countries with a PCV10 NIP or no PCV NIP were not included in this analysis due to the small number of studies per stratum (<5 studies per serotype, age group, PCV type, and PCV period). In countries reporting data from the PCV13 NIP initial period (<3 years duration), serotypes 15B/C in children and 22F in adults were the most frequent. In countries reporting data from the PCV13 NIP later period (≥3 years duration), several PCV20nonPCV13 serotypes were marginally more common for children: 10A (3.5%, 95% confidence interval (CI): 2.8%, 4.4%;), 12F (4.4%; 95% CI: 2.6%, 7.6%), and 15B/C (7.2%; 95% CI: 5.6%, 9.1%), whereas for adults, the most frequent PCV20nonPCV13 serotypes causing IPD were serotypes 8 (10.2%; 95% CI: 7.6%, 13.4%), 12F (6.2%; 95% CI: 4.7–8.1), and 22F (6.7%; 95% CI: 5.7%, 7.8%). Similar patterns were observed when 67 studies with a moderate or serious risk of bias were excluded from the meta-analysis (Table S6).

### 3.5. Incidence of PCV20nonPCV13 Serotypes by Clinical Presentation

Twelve studies conducted in ten countries reported the IR of PCV20nonPCV13 serotypes (one study from a PCV10 NIP country (Austria) and eleven studies from PCV13 NIP countries). Eight studies reported data for children; five reported data for adults; and three reported data for patients of all ages (Table S7). The IRs of individual PCV20nonPCV13 serotype IPD cases varied widely across studies and age groups. Among children, IRs were relatively low for each of the PCV20nonPCV13 serotypes; nonetheless, in most studies serotypes 12F and 15B/C were responsible for the highest IR among clinical presentation. In adults, serotypes 8, 12F, and 22F had the highest IRs across clinical presentations.

### 3.6. Mortality Outcomes by PCV20nonPCV13 Serotype

Nine studies reporting severity outcomes among PCV20nonPCV13 cases were identified [12,16,17,18,19,20,21,22,23] with CFR the most consistently reported outcome (Table 2). CFRs for the individual PCV20nonPCV13 serotypes ranged between 0 and 17% in children, between 0 and 39% in all adults, and between 4 and 39% in adults ≥65 years old. The serotypes associated with the highest CFR were serotypes 10A and 15B/C among children and serotypes 11A and 15B/C in adults or all age groups.

### 3.7. Antimicrobial Non-Susceptibility by PCV20nonPCV13 Serotype

**Figure 3:**
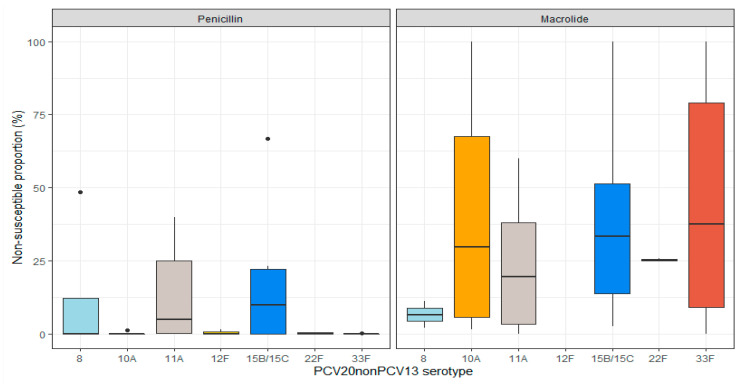
Distribution of penicillin and macrolide non-susceptibility proportions among PCV20nonPCV13 serotypes among all ages. Results with median line only represent either a single study or several studies reporting the same proportions (raw results are available in Table S8). There were no cases of macrolide non-susceptible serotype 12F.

Sixteen studies [16,24,25,26,27,28,29,30,31,32,33,34,35,36,37,38] included in the assessment of antimicrobial non-susceptibility reported the proportion of penicillin (n = 15), macrolide (n = 10), or MDR (n = 3) non-susceptible PCV20nonPCV13 isolates among all ages (Table S8). The non-susceptibility proportion for the PCV20nonPCV13 serotypes individually ranged from 0% to 67% for penicillin, from 0% to 10% for macrolides, and from 0% to 9% for MDR (Figure 3).

### 3.8. Summary of the Characteristics of the PCV20nonPCV13 Serotypes

The characteristics of each PCV20nonPCV13 serotype are summarized in Table 3, based on available data from this review. In children, serotypes 15B/C presented a high proportion, mortality, and antibiotic non-susceptibility. Serotypes 10A and 12F were also frequent causes of pediatric IPD. The most frequent causes of IPD in adults were serotypes 8 (10%), 12F (6%), and 22F (7%), most notably in the PCV13 NIP later period; conversely, serotypes 15B/C and 11A showed higher mortality and antibiotic non-susceptibility rates in adults than serotypes 8, 12F, and 22F, but they were less prevalent among any of the clinical syndromes.

## 4. Discussion

In this systematic review, the proportions of PCV20nonPCV13 or PCV20nonPCV15 serotypes represented around 28% or 20% of IPD, respectively, in children, as well as in adults, among the studies reporting IPD for each of the PCV20nonPCV13 serotypes. These proportions gradually increased over time in countries where these data were available. Our results also demonstrated the proportions of IPD by serotype grouping were relatively similar for children and adults, though the contribution of individual PCV20nonPCV13 serotypes were different between age groups. The proportion of non-sterile site disease due to PCV20nonPCV13 serotypes, which was reported in three studies only, varied from 16% in adults (Non-IPD presentation unspecified) to 29% for pneumococcal AOM in children. The corresponding PCV20nonPCV15 percentages were 11% for any Non-IPD (‘Non-IPD, presentation unspecified’) in adults and 17% for pneumococcal AOM in children.

While previous multicenter studies and literature reviews focused on serotype distribution among IPD cases [9,10], our review provides additional insights on the PCV20nonPCV13 profile: serotype distribution by clinical manifestations (including non-invasive disease) and serotype-specific incidence rates, case fatality rates, and antimicrobial non-susceptibility proportions. This allowed us to describe the characteristics of each PCV20nonPCV13 serotype.

Our results for IPD serotype distribution are similar to those reported by five recently published multicenter studies and reviews [9,10,39,40,41]. The proportion of PCV20nonPCV13 serotypes in IPD was estimated at 38% across all age groups in Europe (2018) by a systematic review and at 29% in children in an analysis of 30 high-income countries [9,10]. A European multicenter study on IPD also showed that the PCV20nonPCV13 serotype proportion increased over time between 2012 and 2018, from 30% to 41% in children and from 30% to 38% in older adults [40]. In that multicenter study, the most frequent PCV20nonPCV13 serotypes in 2018 in children were serotypes 8, 10A, and 12F (7% each) and in adults >65 years-old were serotypes 8 (17%) and 22F (7%) [40]. A worldwide multicenter study found that serotype 15B/C was the most frequent PCV20nonPCV13 serotype causing IPD among children in PCV13 NIP countries (10%), followed by 12F (6%) and 10A (5.5%), while serotypes 8 (9%) and 22F (8%) predominated in adults [41]. Finally, a review of high-income countries identified that the proportion of PCV20nonPCV13 IPD serotypes was higher among countries with ≥3 years of PCV13 use in the NIP than in those with <3 years of use (39.2% vs. 33.6%) [42].

The contribution of individual serotypes and serotype groupings to IPD varied geographically, a finding supported by other systematic reviews and multicenter studies [39,42,43]. For instance, serotype 8 represented <1% of all IPD cases in the United States (2012–13), whereas in the UK (2016–17) this serotype represented 20% of all IPD [42,44,45]. Several hypotheses have been proposed to explain these differences, such as variations in clinical threshold for blood culturing, vaccine schedules, and clonal epidemiology across countries, but this remains to be elucidated [42,46,47].

Our review highlighted a gradual increase over time in the proportion of the PCV20nonPCV13 serotypes overall, and for some individual serotypes, where PCV13 had been introduced in the pediatric NIP. For example, serotype 8 increased in both children and adults (Figure 2). The multicenter European study reported a >2-fold increase in the proportion of IPD due to serotype 8 from 2012 to 2018 in both age groups, which was the major contributor to the observed increase of PCV20nonPCV13 serotypes [41]. In Spain, the proportion of serotype 8 increased between 2015 and 2018, which was due to the expansion of a single clone (ST53) [36] that also predominated in adults in Denmark [48]. Similarly, the pooled proportion of serotype 10A increased in children, and serotype 12F increased in adults. Although increases in the proportions of disease do not necessarily reflect increases in incidence, it is important to note that no clearly dominant PCV20nonPCV13 serotype has been observed post-PCV10/13 vaccine introduction, a difference from the post-PCV7 period when serotype 19A rapidly increased in proportion and incidence [48].

Serotype groupings based only on vaccine composition may mask differences between individual serotypes, in frequency or in clinical characteristics. For example, serotype 12F was the most frequent PCV20nonPCV13 serotype among meningitis cases in children in the initial PCV13 NIP period (11%) and in adults in the later PCV13 NIP period (9%), a finding consistent with the highly invasive disease potential of 12F shown in several studies [49,50]. A previous worldwide multicenter study on pneumococcal meningitis reported also that 12F was among the leading serotypes causing meningitis in ‘mature’ PCV13 NIP programs (i.e., 5 to 7 years after PCV10/13 introduction) [39]. In children, serotype 15B/C was the most common invasive and non-invasive serotype, a finding supported by an earlier systematic review [43]. Serotype 11A was a common cause of pediatric AOM but not IPD.

Our review had several strengths, such as the inclusion of 127 studies, representation of multiple WHO regions, and sufficient robustness (i.e., ‘sensitivity’) to describe the characteristics of each of the PCV20nonPCV13 serotypes. We had several limitations as well. Studies varied in their design, population settings, PCV use, and time since PCV introduction. We attempted to account for this variation by pooling estimates (by age group, PCV13 program duration, and type of clinical outcome) and by using random-effects meta-analysis, as we assumed that the true serotype proportion could vary across studies [51]. For individual serotypes, other than the proportion of IPD due to each serotype, limited data were available, such as for incidence rates, proportion of non-invasive disease, antimicrobial resistance, and severity measures (other than CFR). The duration of PCV use was based on the introduction of PCV13 in the NIP, but some PCV13 use in certain national private markets before the NIP cannot be excluded. PCV13 transitioned from established PCV7 NIPs in the included high-income countries; however, PCV13 private markets were unlikely to be substantial in the few included low- and middle-income countries. Lastly, while we focused on the proportions of IPD due to specific serotypes and serotype groups, these data should be interpreted with caution. Specifically, when evaluating proportions, data can be misinterpreted because either a natural or vaccine-induced decline in the incidence of some serotypes can lead to a reciprocal increase in the proportion of IPD due to remaining serotypes even with no increase in incidence of the latter. In particular, effective PCV programs have reduced PCV serotype disease; thus, even with static incidence, the proportions of non-PCV serotype disease will become larger over time [52]. Future studies of specific serotypes should preferably measure absolute disease rates rather than proportions of disease.

## 5. Conclusions

This SLR of 127 studies (conducted mainly in the Americas, Europe, and Western Pacific WHO regions), which reported data from countries with a PCV13 NIP (n = 112), a PCV10 NIP (n = 10), or without a PCV NIP (n = 5), indicated that the PCV20nonPCV13 serotypes represented around one-third of all IPD in children and adults, that was documented in countries with PCV13 programs to increase over time. These serotypes also caused one-third of pneumococcal AOM in children. To further an understanding of the characteristics of individual serotypes, whenever possible, data tables of clinical and epidemiological characteristics, by serotype, should be reported. Within the PCV20nonPCV13 group, individual serotypes varied in their contribution to pneumococcal morbidity, mortality, and antibiotic non-susceptibility. These results can be used to inform decision-making on the use of PCVs for children and adults in different PCV use settings and populations.

## Figures and Tables

**Table 1:** Pooled proportion (%) of cases due to all seven PCV20nonPCV13 serotypes and all five PCV20nonPCV15 serotypes by age group, source of sample and clinical presentation.

Age Group	Sampling Site and Clinical Presentation	Proportion All PCV20nonPCV13 ^1^		Proportion All PCV20nonPCV15 ^1^	
		n, Studies (Isolates)	Mean (Min–Max)	n, Studies (Isolates)	Mean (Min–Max)
Children		18 (6062)		21 (6615)	
	Sterile site: IPD overall	17 (6006)	27.8 (0–52.0)	19 (6395)	19.7 (0–41.8)
	IPD, presentation specified:				
	Pneumococcal bacteremia	1 (25)	20.0	2 (89)	24.9 (20.0–29.7)
	Pneumococcal meningitis	1 (18)	11.2	2 (50)	16.5 (11.2–21.8)
	Bacteremic pneumococcal pneumonia	1 (187)	3.6	2 (313)	7.3 (3.1–10.2)
	Other IPD ^2^	1 (23)	34.6	1 (23)	26.0
	IPD, presentation unspecified ^3^	16 (5753)	28.3 (0–52.0)	17 (5920)	21.4 (0–41.8)
	Non-sterile site: Non-IPD overall	1 (56)	28.6	2 (220)	16.5 (9.7–23.2)
	Non-IPD, presentation specified:				
	Pneumococcal AOM	1 (56)	28.6	2 (220)	16.5 (9.7–23.2)
Adults		20 (44,349)		23 (49,699)	
	Sterile site: IPD overall	16 (42,323)	27.5 (8.9–55.4)	18 (47,505)	19.7 (8.0–37.3)
	IPD, presentation specified:				
	Pneumococcal bacteremia	1 (101)	26.7	1 (101)	22.7
	Pneumococcal meningitis	1 (98)	27.5	2 (375)	25.7 (20.4–31.0)
	Bacteremic pneumococcal pneumonia	3 (1071)	23.1 (19.9–28.1)	4 (5976)	17.1 (14.6–23.3)
	Other IPD ^2^	1 (51)	25.5	1 (51)	23.5
	IPD, presentation unspecified ^3^	13 (41,002)	28.6 (8.9–55.4)	13 (41,002)	19.6 (8.0–37.3)
	Non-sterile site: Non-IPD overall	2 (312)	15.8 (9.5–22.0)	2 (312)	10.8 (5.7–15.8)
	Non-IPD, presentation specified	2 (312)	15.8 (9.5–22.0)	2 (312)	10.8 (5.7–15.8)
	Undifferentiated site	3 (1714)	14.6 (8.7–23.9)	4 (1882)	8.0 (4.8–17.5)
	All pneumococcal pneumonia	3 (1714)	14.6 (8.7–23.9)	4 (1882)	8.0 (4.8–17.5)
All ages		14 (14,381)		15 (15,924)	
	Sterile site: IPD overall	14 (14,381)	29.1 (0–58.3)	15 (15,924)	21.6 (0–40.8)
	IPD, presentation specified:				
	Pneumococcal bacteremia	1 (2)	0 ^4^	1 (2)	0 ^4^
	Pneumococcal meningitis	3 (2084)	34.8 (29.2–58.3)	3 (2084)	25.0 (23.0–33.3)
	Bacteremic pneumococcal pneumonia	1 (57)	35.1	1 (57)	26.3
	IPD, presentation unspecified ^3^	11 (12,238)	28.4 (10.0–47.8)	12 (13,781)	21.3 (9.3–40.8)

Abbreviations: AOM = acute otitis media; IPD = invasive pneumococcal disease; n = number of studies; min = minimum calculated percentage (only reported if >1 study); max = maximum calculated percentage (only reported if >1 study). ^1^ Proportions based on studies that reported data for all of the PCV20nonPCV13 serotypes (n = 36 studies) or PCV20nonPCV15 serotypes (n = 42 studies). ^2^ Infections of other normally sterile sites (joint fluid, pericardial fluid, peritoneal fluid, and infected bone, among others). ^3^ Refers to cases where the precise clinical presentation was not reported. ^4^ Only four cases were identified in this age group; none were due to the PCV20nonPCV13 or PCV20nonPCV15 serotypes.

**Table 2:** Case fatality ratio (%) by PCV20nonPCV13 serotypes.

Sampling Site	Clinical Presentation	Region	Country	Study Period	Age	Reference	8	10A	11A	12F	15B/C	22F	33F
Children													
Sterile site: IPD	IPD, presentation unspecified ^1^	EUR	UK (England)	2002–2014	<5 y	[21]	nr	nr	nr	nr	nr	3	3
		EUR	UK	2014–2018	<15 y	[12]	1	4	4	5	12	7	nr
		WPR	Republic of Korea	2011–2014	<18 y	[17]	nr	17	0	0	0	nr	0
Adults													
Sterile site: IPD	IPD, presentation unspecified ^1,2^	EUR	France	2014–2017	≥18 y	[19]	5	33	32	14	nr	16	nr
		EUR	Netherlands	2004–2012	≥5 y	[22]	9	14	25	10	21	12	15
		EUR	Spain (Catalonia)	2014–2016	>65 y	[18]	7	14	22	4	22	11	5
		EUR	UK	2002–2014	5–64 y	[21]	6	18	30	14	19	11	10
		EUR	UK	2002–2014	>65 y	[21]	25	38	39	21	24	28	26
		EUR	UK	2014–2018	>65 y	[12]	15	nr	39	18	nr	22	25
Undifferentiated site	All pneumococcal pneumonia	EUR	France	2008–2012	>18 y	[16]	nr	nr	20	0	nr	nr	nr
All ages													
Sterile site: IPD	IPD, presentation specified:												
	Pneumococcal meningitis	EUR	UK	2011–2016	all	[20]	34	nr	nr	nr	nr	nr	nr
	IPD, presentation unspecified ^1^	EUR	UK	2014–2018	all	[12]	9	nr	30	10	nr	16	17

Abbreviations: EUR = Europe; WPR = Western Pacific; nr = not reported. ^1^ Refers to cases where clinical presentation was reported (i.e., IPD), but not the precise sampling site. ^2^ Excluding pneumococcal meningitis.

**Table 3:** Epidemiological characteristics (proportions, case fatality, and non-susceptibility) of the PCV20nonPCV13 serotypes.

Population	Epidemiological Characteristic (Cause of)	Serotype						
		8	10A	11A	12F	15B/C	22F	33F
Children	IPD ^1,2^	++	++	+	++	+++	++	++
	Pneumococcal meningitis ^2,3^	+++	++	+	++	++++	+++	++
	Bacteremic pneumococcal pneumonia ^2,3^	++	++	+	++	++	+	++
	Non-IPD ^2,3^	+	+++	+	+	++++	nr	+
	Pneumococcal AOM ^2,3^	+	+++	+++	+	++++	++	+
Adults	IPD ^1^	++++	+	+	+++	+	+++	+
	Pneumococcal meningitis ^3^	+++	++	++	++++	+	++	+
	Bacteremic pneumococcal pneumonia ^3^	++++	+	+	+++	+	++	+
	Non-IPD ^3^	+	+	+	+	+	+	+
Children	Death due to IPD ^4^	+	++++	++	++	++++	+++	++
Adults	Death due to IPD ^5^	++	+++	++++	++	++++	++	+++
All Ages	Penicillin non-susceptible IPD or Non-IPD ^6^	+	+	+	+	++	+	+
	Macrolide non-susceptible IPD or Non-IPD ^6^	++	+++	+++	nr	++++	+++	++++

Abbreviations: AOM = acute otitis media; CFRs, case fatality reports; CLSI = Clinical and Laboratory Standards Institute; EUCAST = European Committee on Antimicrobial Susceptibility Testing; IPD = invasive pneumococcal disease; nr = not reported. ^1^ According to the pooled proportions (Table S5). +: ≤2%; ++: 3–5%; +++: 6–8%; ++++: >8%. ^2^ In settings with later period PCV13 programs. ^3^ According to the mean proportions per clinical syndrome (Table S4). +: ≤2%; ++: 3–5%; +++: 6–8%; ++++: >8%. ^4^ According to the CFRs per study (Table 2). +: all studies ≤1%; ++: ≥1 study 2–5%; +++: ≥1 study >5%; ++++: all studies >5% or one study >10%. ^5^ According to the CFRs per study (Table 2). +: all studies <20%; ++: at least one study in any adults and/or 65+ ≥20%; +++: most studies ≥20% in any adults and/or 65+; ++++: all studies ≥20%. ^6^ According to the non-susceptibility medians (Figure 3). +: <5%; ++: 5–9%; +++: 10–29%; ++++: ≥30%; according to CLSI or EUCAST criteria.

## Data Availability

Not applicable.

## Supplementary Materials

The following supporting information can be downloaded at https://www.mdpi.com/article/10.3390/microorganisms11071816/s1, Text S1. Search strategy; Table S1. Timeline for PCV use in country and regional NIPs; Table S2. Risk of bias tool; Figure S1: Study selection process: PRISMA flowchart; Figure S2: Distribution of studies by clinical presentation type, age group, and WHO region; Table S3: Description of studies included in the systematic literature review; Table S4: PCV20nonPCV13 serotype-specific proportion (%) by clinical presentation: number of references (n), Median (med.), and minimum and maximum (min–max); Table S5: Meta-analysis of serotype-specific proportion* of IPD isolates: A. Children and B. Adults; Table S6: Sensitivity analysis restricted to studies without moderate or serious risk of bias: Subset meta-analysis of serotype-specific proportion* of IPD isolates: A. Children B. Adults; Table S7. Incidence rate of PCV20nonPCV13 serotypes (per 100,000 person-years if not stated otherwise); Table S8: Proportion (%) of PCV20nonPCV13 serotypes non-susceptible to penicillin or a macrolide or that were multi-drug resistant (MDR).

## References

[1] Papadatou I., Tzovara I., Licciardi P.V. (2019). The role of serotype-specific immunological memory in pneumococcal vaccination: Current knowledge and future prospects. Vaccines.

[2] Bonten M.J., Huijts S.M., Bolkenbaas M., Webber C., Patterson S., Gault S., van Werkhoven C.H., van Deursen A.M., Sanders E.A., Verheij T.J. (2015). Polysaccharide conjugate vaccine against pneumococcal pneumonia in adults. N. Engl. J. Med..

[3] Dagan R., Van Der Beek B.A., Ben-Shimol S., Pilishvili T., Givon-Lavi N. (2021). Effectiveness of the 7- and 13-valent pneumococcal conjugate vaccines against vaccine-serotype otitis media. Clin. Infect. Dis..

[4] Hurley D., Griffin C., Young M., Scott D.A., Pride M.W., Scully I.L., Ginis J., Severs J., Jansen K.U., Gruber W.C. (2021). Safety, tolerability, and immunogenicity of a 20-valent pneumococcal conjugate vaccine (PCV20) in adults 60 to 64 years of age. Clin. Infect. Dis..

[5] Bonnave C., Mertens D., Peetermans W., Cobbaert K., Ghesquiere B., Deschodt M., Flamaing J. (2019). Adult vaccination for pneumococcal disease: A comparison of the national guidelines in Europe. Eur. J. Clin. Microbiol. Infect. Dis..

[6] Senders S., Klein N.P., Lamberth E., Thompson A., Drozd J., Trammel J., Peng Y., Giardina P.C., Jansen K.U., Gruber W.C. (2021). Safety and Immunogenicity of a 20-valent Pneumococcal Conjugate Vaccine in Healthy Infants in the United States. Pediatr. Infect. Dis. J..

[7] van Selm S., van Cann L.M., Kolkman M.A., van der Zeijst B.A., van Putten J.P. (2003). Genetic basis for the structural difference between Streptococcus pneumoniae serotype 15B and 15C capsular polysaccharides. Infect. Immun..

[8] Higgins J.P., Thompson S.G. (2002). Quantifying heterogeneity in a meta-analysis. Stat. Med..

[9] Janssens E., Flamaing J., Vandermeulen C., Peetermans W.E., Desmet S., De Munter P. (2023). The 20-valent pneumococcal conjugate vaccine (PCV20): Expected added value. Acta Clin. Belg..

[10] Grant L.R., Slack M.P., Theilacker C., Vojicic J., Dion S., Reinert R.R., Jodar L., Gessner B.D. (2023). Distribution of serotypes causing invasive pneumococcal disease in children from high-income countries and the impact of pediatric pneumococcal vaccination. Clin. Infect. Dis..

[11] Von Elm E., Altman D.G., Egger M., Pocock S.J., Gøtzsche P.C., Vandenbroucke J.P. (2007). The Strengthening the Reporting of Observational Studies in Epidemiology (STROBE) statement: Guidelines for reporting observational studies. Bull. World Health Organ..

[12] Amin-Chowdhury Z., Collins S., Sheppard C., Litt D., Fry N.K., Andrews N., Ladhani S.N. (2020). Characteristics of invasive pneumococcal disease caused by emerging serotypes after the introduction of the 13-valent pneumococcal conjugate vaccine in England: A prospective observational cohort study, 2014–2018. Clin. Infect. Dis..

[13] World Health Organization Pneumococcus: Vaccine-Preventable Diseases Surveillance Standards. https://www.who.int/publications/m/item/vaccine-preventable-diseases-surveillance-standards-pneumococcus.

[14] Hao L., Kuttel M.M., Ravenscroft N., Thompson A., Prasad A.K., Gangolli S., Tan C., Cooper D., Watson W., Liberator P. (2022). Streptococcus pneumoniae serotype 15B polysaccharide conjugate elicits a cross-functional immune response against serotype 15C but not 15A. Vaccine.

[15] Peterson J., Welch V., Losos M., Tugwell P. (2011). The Newcastle-Ottawa Scale (NOS) for Assessing the Quality of Nonrandomised Studies in Meta-Analyses.

[16] Bedos J.P., Varon E., Porcher R., Asfar P., Le Tulzo Y., Megarbane B., Mathonnet A., Dugard A., Veinstein A., Ouchenir K. (2018). Host-pathogen interactions and prognosis of critically ill immunocompetent patients with pneumococcal pneumonia: The nationwide prospective observational STREPTOGENE study. Intensive Care Med..

[17] Cho E.Y., Choi E.H., Kang J.H., Kim K.H., Kim D.S., Kim Y.J., Ahn Y.M., Eun B.W., Oh S.H., Cha S.H. (2016). Early Changes in the Serotype Distribution of Invasive Pneumococcal Isolates from Children after the Introduction of Extended-valent Pneumococcal Conjugate Vaccines in Korea, 2011–2013. J. Korean Med. Sci..

[18] Ciruela P., Broner S., Izquierdo C., Pallarés R., Muñoz-Almagro C., Hernández S., Grau I., Domínguez A., Jané M. (2019). Indirect effects of paediatric conjugate vaccines on invasive pneumococcal disease in older adults. Int. J. Infect. Dis..

[19] Danis K., Varon E., Lepoutre A., Janssen C., Forestier E., Epaulard O., N’Guyen Y., Labrunie A., Lanotte P., Gravet A. (2019). Factors Associated with Severe Nonmeningitis Invasive Pneumococcal Disease in Adults in France. Open Forum. Infect. Dis..

[20] Oligbu G., Collins S., Djennad A., Sheppard C.L., Fry N.K., Andrews N.J., Borrow R., Ramsay M.E., Ladhani S.N. (2019). Effect of Pneumococcal Conjugate Vaccines on Pneumococcal Meningitis, England and Wales, July 1, 2000–June 30, 2016. Emerg. Infect. Dis..

[21] van Hoek A.J., Andrews N., Waight P.A., George R., Miller E. (2012). Effect of serotype on focus and mortality of invasive pneumococcal disease: Coverage of different vaccines and insight into non-vaccine serotypes. PLoS ONE.

[22] Wagenvoort G.H., Sanders E.A., Vlaminckx B.J., Elberse K.E., de Melker H.E., van der Ende A., Knol M.J. (2016). Invasive pneumococcal disease: Clinical outcomes and patient characteristics 2–6 years after introduction of 7-valent pneumococcal conjugate vaccine compared to the pre-vaccine period, the Netherlands. Vaccine.

[23] Rudnick W., Liu Z., Shigayeva A., Low D.E., Green K., Plevneshi A., Devlin R., Downey J., Katz K., Kitai I. (2013). Pneumococcal vaccination programs and the burden of invasive pneumococcal disease in Ontario, Canada, 1995–2011. Vaccine.

[24] Correa M., Onieva-García M.Á., López I., Montiel N. (2018). Invasive neumococcal disease in Costa del Sol Hospital: Replacement by non-vaccinable serotypes. Rev. Esp. Salud Publica.

[25] Fenoll A., Aguilar L., Gimenez M.J., Vicioso M.D., Robledo O., Granizo J.J., Coronel P. (2012). Variations in serotypes and susceptibility of adult non-invasive *Streptococcus pneumoniae* isolates between the periods before (May 2000–May 2001) and 10 years after (May 2010–May 2011) introduction of conjugate vaccines for child immunisation in Spain. Int. J. Antimicrob. Agents.

[26] Golden A.R., Adam H.J., Zhanel G.G. (2016). Invasive Streptococcus pneumoniae in Canada, 2011–2014: Characterization of new candidate 15-valent pneumococcal conjugate vaccine serotypes 22F and 33F. Vaccine.

[27] Izquierdo C., Ciruela P., Hernández S., García-García J.J., Esteva C., Moraga-Llop F., Díaz-Conradi A., Martínez-Osorio J., Solé-Ribalta A., de Sevilla M.F. (2020). Pneumococcal serotypes in children, clinical presentation and antimicrobial susceptibility in the PCV13 era. Epidemiol. Infect..

[28] Kawaguchiya M., Urushibara N., Aung M.S., Shinagawa M., Takahashi S., Kobayashi N. (2017). Serotype distribution, antimicrobial resistance and prevalence of pilus islets in pneumococci following the use of conjugate vaccines. J. Med. Microbiol..

[29] Ktari S., Jmal I., Mroua M., Maalej S., Ben Ayed N.E., Mnif B., Rhimi F., Hammami A. (2017). Serotype distribution and antibiotic susceptibility of *Streptococcus pneumoniae* strains in the south of Tunisia: A five-year study (2012–2016) of pediatric and adult populations. Int. J. Infect. Dis..

[30] Mendes R.E., Hollingsworth R.C., Costello A., Jones R.N., Isturiz R.E., Hewlett D., Farrell D.J. (2015). Noninvasive Streptococcus pneumoniae serotypes recovered from hospitalized adult patients in the United States in 2009 to 2012. Antimicrob. Agents Chemother..

[31] Méndez-Lage S., Losada-Castillo I., Agulla-Budiño A. (2015). Streptococcus pneumoniae: Serotype distribution, antimicrobial susceptibility, risk factors and mortality in Galicia over a two year-period. Enferm. Infecc. Microbiol. Clin..

[32] Naziat H., Saha S., Islam M., Saha S., Uddin M.J., Hussain M., Luby S.P., Darmstadt G.L., Whitney C.G., Gessner B.D. (2018). Epidemiology of otitis media with otorrhea among Bangladeshi children: Baseline study for future assessment of pneumococcal conjugate vaccine impact. Pediatr. Infect. Dis. J..

[33] Richter S.S., Heilmann K.P., Dohrn C.L., Riahi F., Diekema D.J., Doern G.V. (2013). Pneumococcal serotypes before and after introduction of conjugate vaccines, United States, 1999–2011. Emerg. Infect. Dis..

[34] Sanz J.C., Rodríguez-Avial I., Ríos E., García-Comas L., Ordobás M., Cercenado E. (2020). Increase of serotype 8, ST53 clone, as the prevalent strain of Streptococcus pneumoniae causing invasive disease in Madrid, Spain (2012–2015). Enferm. Infecc. Microbiol. Clin..

[35] Sheppard C., Fry N.K., Mushtaq S., Woodford N., Reynolds R., Janes R., Pike R., Hill R., Kimuli M., Staves P. (2016). Rise of multidrug-resistant non-vaccine serotype 15A Streptococcus pneumoniae in the United Kingdom, 2001 to 2014. Euro Surveill..

[36] Suaya J.A., Mendes R.E., Sings H.L., Arguedas A., Reinert R.-R., Jodar L., Isturiz R.E., Gessner B.D. (2020). Streptococcus pneumoniae serotype distribution and antimicrobial nonsusceptibility trends among adults with pneumonia in the United States, 2009–2017. J. Infect..

[37] Uddén F., Rünow E., Slotved H.C., Fuursted K., Ahl J., Riesbeck K. (2021). Characterization of Streptococcus pneumoniae detected in clinical respiratory tract samples in southern Sweden 2 to 4 years after introduction of PCV13. J. Infect..

[38] Nakano S., Fujisawa T., Ito Y., Chang B., Suga S., Noguchi T., Yamamoto M., Matsumura Y., Nagao M., Takakura S. (2016). Serotypes, antimicrobial susceptibility, and molecular epidemiology of invasive and non-invasive Streptococcus pneumoniae isolates in paediatric patients after the introduction of 13-valent conjugate vaccine in a nationwide surveillance study conducted in Japan in 2012–2014. Vaccine.

[39] Garcia Quesada M., Yang Y., Bennett J.C., Hayford K., Zeger S.L., Feikin D.R., Peterson M.E., Cohen A.L., Almeida S.C., Ampofo K. (2021). Serotype distribution of remaining pneumococcal meningitis in the mature PCV10/13 period: Findings from the PSERENADE project. Microorganisms.

[40] Hanquet G., Krizova P., Dalby T., Ladhani S.N., Nuorti J.P., Danis K., Mereckiene J., Knol M.J., Winje B.A., Ciruela P. (2022). Serotype Replacement after Introduction of 10-Valent and 13-Valent Pneumococcal Conjugate Vaccines in 10 Countries, Europe. Emerg. Infect. Dis..

[41] Quesada M.G., Hetrich M., Knoll M.D. (2021). 1181. Serotype Distribution by Age of Remaining Invasive Pneumococcal Disease after Long-Term PCV10/13 Use: The PSERENADE Project. Open Forum Infect. Dis..

[42] Lewnard J.A., Hanage W.P. (2019). Making sense of differences in pneumococcal serotype replacement. Lancet Infect. Dis..

[43] Balsells E., Guillot L., Nair H., Kyaw M.H. (2017). Serotype distribution of *Streptococcus pneumoniae* causing invasive disease in children in the post-PCV era: A systematic review and meta-analysis. PLoS ONE.

[44] Moore M.R., Link-Gelles R., Schaffner W., Lynfield R., Lexau C., Bennett N.M., Petit S., Zansky S.M., Harrison L.H., Reingold A. (2015). Effect of use of 13-valent pneumococcal conjugate vaccine in children on invasive pneumococcal disease in children and adults in the USA: Analysis of multisite, population-based surveillance. Lancet Infect. Dis..

[45] Ladhani S.N., Collins S., Djennad A., Sheppard C.L., Borrow R., Fry N.K., Andrews N.J., Miller E., Ramsay M.E. (2018). Rapid increase in non-vaccine serotypes causing invasive pneumococcal disease in England and Wales, 2000–2017: A prospective national observational cohort study. Lancet Infect. Dis..

[46] Hausdorff W.P. (2019). Pneumococcal conjugate vaccines in different settings. Lancet Infect. Dis..

[47] Miller E., Andrews N.J., Waight P.A., Slack M.P., George R.C. (2011). Herd immunity and serotype replacement 4 years after seven-valent pneumococcal conjugate vaccination in England and Wales: An observational cohort study. Lancet Infect. Dis..

[48] Løchen A., Croucher N.J., Anderson R.M. (2020). Divergent serotype replacement trends and increasing diversity in pneumococcal disease in high income settings reduce the benefit of expanding vaccine valency. Sci. Rep..

[49] Yildirim I., Hanage W.P., Lipsitch M., Shea K.M., Stevenson A., Finkelstein J., Huang S.S., Lee G.M., Kleinman K., Pelton S. (2010). Serotype specific invasive capacity and persistent reduction in invasive pneumococcal disease. Vaccine.

[50] Varon E., Cohen R., Béchet S., Doit C., Levy C. (2015). Invasive disease potential of pneumococci before and after the 13-valent pneumococcal conjugate vaccine implementation in children. Vaccine.

[51] Riley R.D., Higgins J.P., Deeks J.J. (2011). Interpretation of random effects meta-analyses. BMJ.

[52] Whitney C.G., Toscano C.M. (2021). Direct effects of pneumococcal conjugate vaccines among children in Latin America and the Caribbean. Lancet Infect. Dis..

