# Correction: Méroc et al. Systematic Literature Review of the Epidemiological Characteristics of Pneumococcal Disease Caused by the Additional Serotypes Covered by the 20-Valent Pneumococcal Conjugate Vaccine. *Microorganisms* 2023, *11*, 1816

**DOI:** 10.3390/microorganisms13040793

**Published:** 2025-03-31

**Authors:** Estelle Méroc, Mark A. Fletcher, Germaine Hanquet, Mary P. E. Slack, Marc Baay, Kyla Hayford, Bradford D. Gessner, Lindsay R. Grant

**Affiliations:** 1 P95 Epidemiology & Pharmacovigilance, Koning Leopold III-laan 1, 3001 Leuven, Belgium; estelle.meroc@p-95.com (E.M.); germaine.hanquet@p-95.com (G.H.); marc.baay@p-95.com (M.B.); 2 Emerging Markets Medical Affairs, Vaccines, Pfizer, 23–25 Av. du Dr Lannelongue, 75014 Paris, France; mark.a.fletcher@pfizer.com; 3 School of Medicine & Dentistry, Griffith University Gold Coast Campus, Parklands Drive, Southport, QLD 4222, Australia; mary.slack@pfizer.com; 4 Medical Development and Scientific Clinical Affairs, Pfizer Vaccines, 500 Arcola Road, Collegeville, PA 19426, USA; kyla.hayford@pfizer.com (K.H.); bradford.gessner@pfizer.com (B.D.G.)

In the original publication [1], there was a mistake in Table S5 (adults), which was a duplicate of Table S5 (children). The corrected Table S5 appears below. The authors state that the scientific conclusions are unaffected. This correction was approved by the Academic Editor. The original publication has also been updated.

## Figures and Tables

**Table S5:** Meta-analysis of serotype-specific proportion * of IPD isolates from children and adults.

Children	Serotype	N (Studies)	Proportion (95% CI)
**PCV13 NIP, initial period (<3 years)**			
	**All PCV13**	26	50.3 (41.3–59.3)
**PCV20nonPCV13**			
	**8**	22	2.1 (1.3–3.3)
	**10A**	24	3.5 (2.8–4.4)
	**11A**	20	1.9 (1.3–2.9)
	**12F**	24	4.4 (2.6–7.6)
	**15B/C**	31	7.2 (5.6–9.1)
	**22F**	25	3.6 (2.5–5.3)
	**33F**	23	3.2 (2.3–4.6)
**PCV13 NIP, later period (≥3 years)**			
	**All PCV13**	17	44.5 (30.6–59.3)
**PCV20nonPCV13**			
	**8**	19	3.8 (2.5–5.8)
	**10A**	18	5.9 (4.5–7.7)
	**11A**	15	1.9 (1.6–2.3)
	**12F**	20	4.9 (3.2–7.4)
	**15B/C**	18	6.2 (4.4–8.7)
	**22F**	20	4.2 (2.7–6.3)
	**33F**	18	4.4 (3.0–6.4)
**Adults**	**Serotype**	**N (Studies)**	**Proportion (95% CI)**
**PCV13 NIP, initial period (<3 years)**			
	**All PCV13**	13	49.5 (45.0–57.0)
	**PCV20nonPCV13**		
	**8**	10	4.1 (1.6–10.4)
	**10A**	8	1.8 (1.0–3.5)
	**11A**	10	3.7 (2.4–5.6)
	**12F**	7	2.4 (1.5–4.0)
	**15B/C**	11	2.5 (1.7–3.6)
	**22F**	13	7.7 (5.9–9.9)
	**33F**	7	1.8 (1.2–2.7)
**PCV13 NIP, later period (≥3 years)**			
	**All PCV13**	9	27.3 (19.6–36.7)
	**PCV20nonPCV13**		
	**8**	13	10.2 (7.6–13.4)
	**10A**	13	2.4 (1.9–3.0)
	**11A**	13	2.7 (2.4–3.1)
	**12F**	13	6.2 (4.7–8.1)
	**15B/C**	11	1.5 (1.2–2.0)
	**22F**	13	6.7 (5.7–7.8)
	**33F**	12	2.5 (1.8–3.4)

* Calculated with a binomial random-effects meta-analytic model for each serotype-specific proportion independently. Consequently, the sum of all PCV20nonPCV13 serotypes may exceed 100%.
