# Supplementary material for: Systematic Literature Review of the Epidemiological Characteristics of Pneumococcal Disease Caused by the Additional Serotypes Covered by the 20-Valent Pneumococcal Conjugate Vaccine
Source: Microorganisms. 2023 Jul 15;11(7):1816. doi: 10.3390/microorganisms11071816 (PMC10383425; doi:10.3390/microorganisms11071816)
Supplement: Supplementary file 1 [file microorganisms-11-01816-s001.zip › microorganisms-2394856-supplementary.pdf]

Supplementary Materials for

Epidemiological characteristics of pneumococcal disease caused by the additional serotypes covered by the 20-valent pneumococcal conjugate vaccine

Estelle Méroc, Mark A. Fletcher, Germaine Hanquet, Mary P. E. Slack, Marc Baay, Kyla Hayford, Bradford D. Gessner, and Lindsay R. Grant*

Included Materials:

Text S1. Search strategy
Table S1. Timeline for PCV use in country and regional NIPs
Table S2. Risk of bias tool
Figure S1. Study selection process: PRISMA flowchart.
Figure S2. Distribution of studies by clinical presentation type, age group, and WHO region.
Table S3. Description of studies included in the systematic literature review.
Table S4. PCV20nonPCV13 serotype-specific proportion (%) by clinical presentation: number of references (n), median (med.), and minimum and maximum (min–max).
Table S5. Meta-analysis of serotype-specific proportion* of IPD isolates: a. Children and b. Adults.
Table S6. Sensitivity analysis restricted to studies without moderate or serious risk of bias: Subset meta-analysis of serotype-specific proportion* of IPD isolates: a. Children b. Adults.
Table S7. Incidence rate of PCV20nonPCV13 serotypes per 100,000 person-years (if not stated otherwise).
Table S8. Proportion (%) of PCV20nonPCV13 serotypes non-susceptible to penicillin or a macrolide or that were multi-drug resistant (MDR)

Text S1. Search strategy

("2010/01/01" [Date – Publication]: "3000"[Date – Publication]) AND (pneumococ* OR streptococcus pneumoniae) AND (PPSV23 OR PCV10 OR PCV13 OR serotyp*) AND (invasiv* OR noninvasive OR "pneumonia" OR "otitis media" OR (empyema) OR (rhinosinusitis) OR (sinusitis) OR (peritonitis) OR (osteoarticular infection) OR (hemolytic uremic syndrome) OR (septic arthritis) OR (endocarditis)) NOT ("immunogenicity" OR "immune response") AND ((serotype) AND ((urin*) OR (antigen) OR (detection)))

Table S1. Timeline for PCV use in country and regional NIPs

Country	PCV7	PCV10	PCV13
**Argentina**			2012
**Australia**	2005		2010
**Austria**		2012	
**Belgium**	2007	2015/2016	2011 (and 2019)
**Bengladesh**		2015	
**Brazil**		2010/2016	
**Cambodia**			2015
**Canada**			
**Alberta**	2002		2010
**Quebec**	2004	2009	2011
**Ontario**		2009	2010
**China**	NA	NA	NA
**Denmark**	2007		2010
**Ethiopia**		2011	
**France**	2006		2010
**Gambia**	2007		2011
**Germany**	2006	2009 (<10%)	2009
**Hong Kong**	2009	2010	2011
**India**	N/A	N/A	N/A
**Iran**	NA	NA	NA
**Israel**	2010		2012
**Italy**	2005-2007		2010-2012
**Japan**	2011		2013
**Kuwait**	2006		2010
**Malawi**			2011
**Mozambique**		2013	
**Netherlands**	2006	2011	
**Norway**	2006		2011
**Oman**	2008	2010	2012
**Portugal**	2001	2009	2010
**South Africa**	2009		2011
**South Korea**			2010
**Spain**			
**Catalonia**	2006*	(2010:<5%)	2010
**Galicia**			2010
**Madrid**	2006		2010†
**Navarra**	2006*	(2010:<1%)	2010‡
**Sweden**	2009	2010 (~50%)	2010 (~50%)
**Taiwan**	2005		2015
**Tunisia**	NA	NA	NA
**Turkey**	2008		2011
**UK**	2006		2010
**USA**	2000		2010

Abbreviations: NA = no PCV in NIP; NIP = national immunization program; PCV = pneumococcal conjugate vaccine.
* Not universal but recommended and uptake around or above 50%.
† Not universal during 2012–2014 but recommended and uptake >75%.
‡ Not universal during 2010–2015 but recommended and uptake >70%.

Table S2. Risk of bias tool

Domain	Category	Risk of bias
1. Sample size	At least 100 isolates	Low
	Less than 100 isolates	High
2. Study population	All participants had equal opportunity to be in the study	Low
	Not all participants had equal opportunity to be included in the study, e.g., patients from specific risk group or severity level were excluded	High
	No description of the study participants	High
3. Study period	More than 1 year	Low
	Less or equal to 1 year	High
4. Serotyping method	Complete serotyping methods given	Low
	Incomplete serotyping methods given	High
	Not reported	High
5. Non-typed isolates proportion	At least 80% of isolates typed	Low
	Less than 80% of isolates typed	High
6. Outcome (proportion, incidence…)	Both numerator and denominator reported	Low
	Missing data	High

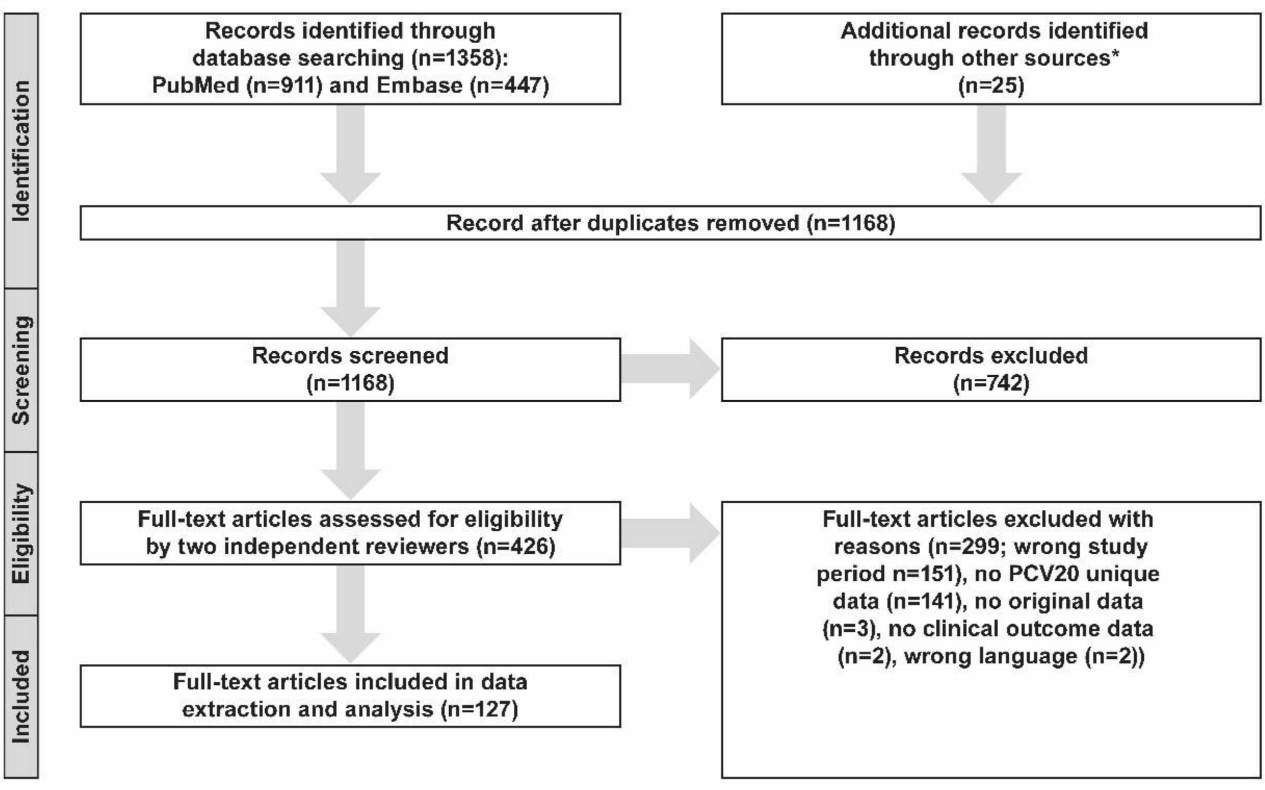

Figure S1. Study selection process: PRISMA flowchart. Proportion of PCV20nonPCV13 and PCV20nonPCV15 serotypes by clinical presentation. *Records identified through reference list checking.

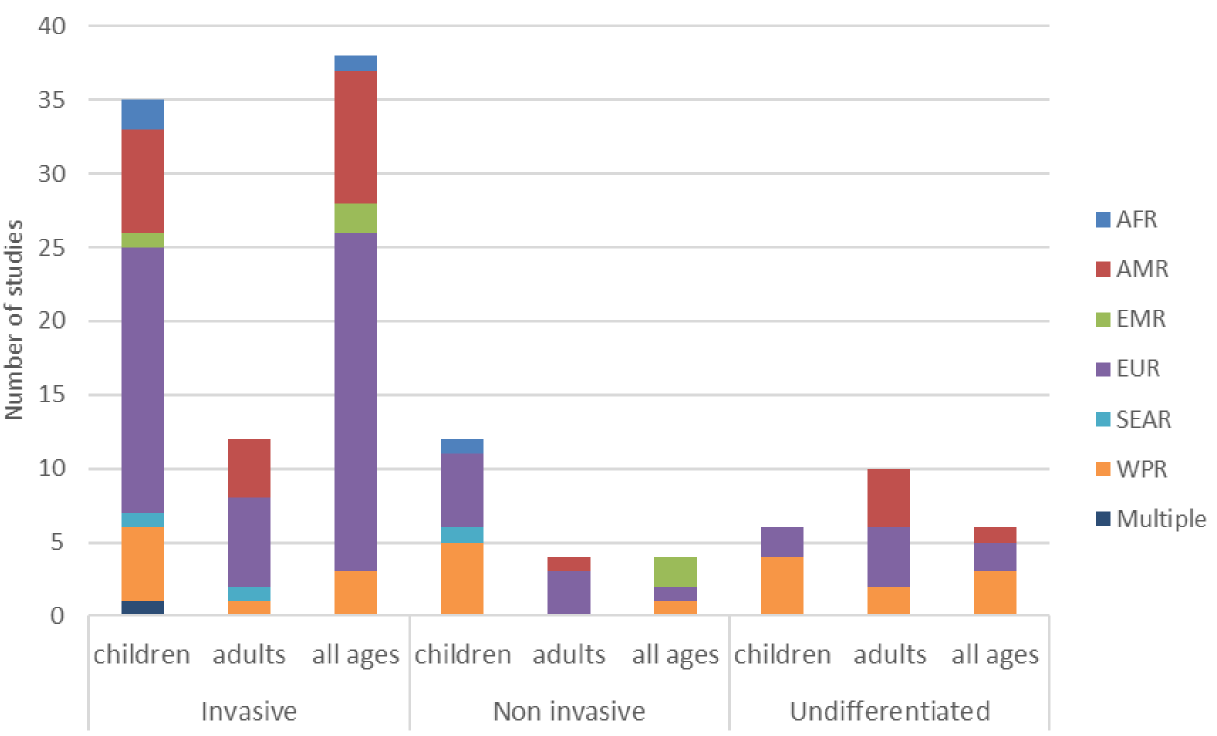

Figure S2. Distribution of studies by clinical presentation type, age group, and WHO region. WHO regions are defined as: AFR, African; AMR, the Americas; EMR, Eastern Mediterranean; EUR, European; SEAR, South-East Asian; WPR, Western Pacific.

Table S3: Description of studies included in the systematic literature review

WHO Region	Country	Reference	Setting	Period	Population	Disease	Outcome
	Portugal	[1]	national	2008 – 2012	<18y	IPD overall	Proportion
	France	[2]	national	2010 – 2014	all ages	meningitis	Proportion
EMR	Oman	[3]	national	2014 – 2016	all ages	IPD overall	Proportion
	UK	[4]	national	2014 – 2018	all ages	IPD overall, bacteremia, other IPD, meningitis, bact. pneumonia	Proportion, Severity
	France	[5]	national	2010 – 2012	<15y	all pneumonia	Proportion
	France	[6]	national	2008 – 2012	>18y	all pneumonia	AMR proportion, Severity, Proportion
	Denmark	[7]	national	2011 – 2011	>15y	bacteremic pneumonia, non-IPD	Proportion
	Israel (Southern)	[8]	regional	2010 – 2013	<2y	AOM	Incidence
	Israel	[9]	national	2010 – 2013	<5y	IPD overall	Incidence
	Israel	[10]	national	2000 – 2016	<5y	bacteremic pneumonia, meningitis, other IPD	Incidence
	UK	[11]	hospital	2008 – 2010	>=16y	non-IPD	Proportion
	US (Alaska)	[12]	regional	2010 – 2013	all ages	IPD overall	Incidence, Proportion
	Canada (Calgary)	[13]	regional	2010 – 2013	>18y; comorbidities	IPD overall	Proportion
	Turkey	[14]	national	2011 – 2014	<18y	IPD overall	Proportion
	Turkey	[15]	national	2015 – 2018	<18y	meningitis, IPD overall, bacteremia	Proportion
	Taiwan	[16]	hospital	2011 – 2015	all ages	all pneumonia	AMR proportion
	Taiwan	[17]	hospital	2011 – 2015	>18y	IPD overall	Proportion
	South Korea	[18]	national	2011 – 2014	<18y	IPD overall	Severity, Proportion
	Taiwan (Northern)	[19]	regional	2010 – 2015	<18y	IPD overall, non-IPD	Proportion
	South Korea	[20]	hospital	2010 – 2013	>19y	all pneumonia	Proportion
WPR	South Korea	[21]	hospital	2015 – 2015	15-91y	IPD + non-IPD	Proportion, AMR proportion

Spain (Catalonia)	[22]	regional	2014 – 2016	>65y	IPD overall	Incidence, Severity
S.Africa	[23]	national	2012 – 2014	<5y; HIV	IPD overall	Proportion
Spain	[24]	hospital	2012 – 2017	0-92y	IPD overall, bacteremic pneumonia, meningitis, bacteremia	Proportion, AMR proportion
France	[25]	national	2014 – 2017	≥18y	IPD overall	Severity, Proportion
Canada	[26]	national	2010 – 2012	all ages	IPD overall	Proportion, AMR proportion
Belgium	[27]	national	2017 – 2018	<16y	IPD overall	Proportion
UK (England and Wales)	[28]	national	2012 – 2017	>65y	IPD overall	Proportion, Incidence
Brazil	[29]	national	2010 – 2012	<5y	IPD overall	Proportion
Spain	[30]	national	2013 – 2015	6mo-6y	non-IPD	Proportion
Spain	[31]	national	2010 – 2011	>18y	non-IPD	AMR proportion, Proportion
China	[32]	hospital	2016 – 2016	<2y	AOM	Proportion
Sweden (Stockholm)	[33]	regional	2011 – 2014	all ages	IPD overall	Incidence, Proportion
Argentina	[34]	national	2012 – 2014	<15y	bacteremic pneumonia	Proportion
Germany	[35]	national	2010 – 2018	<18y	complicated pneumonia	Proportion
Canada	[36]	national	2011 – 2014	all ages	IPD overall	AMR proportion, Proportion
Spain (Navarre)	[37]	regional	2010 – 2013	all ages	IPD overall	Proportion
Australia (Queensl./N.Terr.)	[38]	regional	2005 – 2015	<5y	non-IPD	NA, Proportion
Spain (Catalonia)	[39]	regional	2012 – 2015	<18y	IPD overall	Proportion
Portugal	[40]	national	2012 – 2015	>18y	non-IPD	Proportion
Iran	[41]	hospital	2013 – 2016	<5y	IPD overall	Proportion
Germany	[42]	national	2010 – 2013	all ages	meningitis	Proportion
US	[43]	national	2013 – 2016	≥18y	all pneumonia	Proportion
Spain	[44]	hospital	2012 – 2016	≤17y	meningitis, other IPD, bact.pneumonia, IPD overall, bacteremia	Proportion, AMR proportion

	India	[45]	hospital	2007 – 2017	>18y	IPD overall	Proportion
	Australia	[46]	national	2014 – 2014	all ages	IPD overall	Incidence
	US	[47]	national	2010 – 2011	<18y	meningitis, bact. pneumonia, IPD overall, other IPD, bacteremia	Proportion
AMR	US	[48]	national	2014 – 2017	<18y	IPD overall	Proportion
	Japan (Northern Japan)	[49]	regional	2013 – 2014	all ages	non-IPD	Proportion
	Japan (Hokkaido)	[50]	regional	2016 – 2016	<16y	non-IPD	Proportion
	Japan	[51]	hospital	2011 – 2016	all ages	IPD + non-IPD	Proportion, AMR proportion
	US (Utah)	[52]	regional	2010 – 2012	>18y	IPD overall	Proportion
	Tunisia	[53]	hospital	2012 – 2016	all ages	IPD + non-IPD	Proportion, AMR proportion
	UK (England and Wales)	[54]	national	2016 – 2017	all ages	IPD overall	Proportion, Incidence
	Spain (Madrid)	[55]	regional	2010 – 2015	all ages	IPD overall	Proportion
	Australia	[56]	hospital	1997 – 2016	7-20m	other IPD	Proportion
	Australia (N.Territory)	[57]	regional	2010 – 2013	0-6y	AOM	Proportion
	Canada	[58]	national	2011 – 2015	>16y	all pneumonia	Proportion
	Canada	[59]	national	2010 – 2015	>16y	all pneumonia	Proportion
	Israel (Southern)	[60]	regional	2005 – 2014	3-36mo	bacteremia	Proportion
	Canada (Nunavik)	[61]	regional	2010 – 2016	<5y	IPD overall	Proportion
	France	[62]	national	2012 – 2012	<14y	IPD overall	incidence
	France	[63]	national	2010 – 2012	<16y	meningitis	Proportion
	France	[64]	national	2015 – 2018	3mo-15y	AOM	Proportion
	Germany	[65]	national	2010 – 2017	<18y	complicated pneumonia	Proportion
	Sweden	[66]	hospital	2011 – 2014	all ages	IPD overall	Proportion
Multiple	Hong Kong, Israel, Malawi, S.Africa	[67]	multi-national	NA – NA	<3y	IPD overall	Proportion
	UK (England and Wales)	[68]	national	2010 – 2016	3-59mo	IPD overall, meningitis, other IPD, IPD and LRTI	Severity, Proportion

SEAR	Bangladesh	[69]	hospital	2013 – 2015	<15y; neph. syndr.	other IPD	Proportion
	US (North. California)	[70]	regional	1996 – 2011	>18y; HIV	IPD overall	Proportion
	Canada (North.Alberta)	[71]	regional	2000 – 2014	all ages; asplania	IPD overall	Proportion
	Mozambique (Southern)	[72]	regional	2012 – 2015	<5y	IPD overall	Proportion
	US	[73]	national	2010 – 2012	>18y	non-IPD	Proportion, AMR proportion
	Spain (Galicia)	[74]	regional	2011 – 2012	all ages	IPD overall	AMR proportion, Proportion
	Spain	[75]	hospital	2011 – 2014	>18y	all pneumonia	Proportion
	Kuwait	[76]	national	2006 – 2014	all ages	IPD and LRTI, IPD overall	Proportion
	Kuwait	[77]	national	2010 – 2013	all ages	IPD overall	AMR proportion, Proportion
	UK (Oxfordshire)	[78]	regional	1996 – 2013	all ages	IPD overall	Incidence, Proportion
	US	[79]	national	2010 – 2013	all ages	IPD overall	Proportion
	Japan	[80]	national	2012 – 2014	2mo-15y	IPD overall, IPD + non-IPD, non-IPD	Proportion, AMR proportion
	Bangladesh	[81]	hospital	2014 – 2015	<18y	AOM	AMR proportion, Proportion
AFR	Ethiopia	[82]	hospital	2016 – 2017	<15y	AOM	Proportion
	Japan	[83]	hospital	2011 – 2014	>18y	all pneumonia	Proportion
	Spain (South. Catalonia)	[84]	regional	2010 – 2013	<14y	AOM	Proportion
	US	[85]	national	2000 – 2014	<18y; transplant	IPD overall	Proportion
	US	[86]	hospital	2005 – 2015	<60d	IPD overall	Proportion
	US	[87]	national	2010 – 2015	<2mo	IPD overall	Proportion
	UK	[88]	national	2011 – 2016	all ages	meningitis, IPD overall	NA, Severity, Proportion
	France	[89]	national	2001 – 2017	all ages	IPD overall	Proportion
	UK	[90]	hospital	2013 – 2018	>16y	non-IPD, IPD overall, all pneumonia	Incidence, Proportion

	Brazil (South-Eastern)	[91]	regional	1990 – 2014	all ages	IPD overall	Proportion
	Italy (Apulia)	[92]	regional	2013 – 2015	>65y	non-IPD	Proportion
	US	[93]	national	2010 – 2011	all ages	IPD overall, non-IPD	Proportion, AMR proportion
	Austria	[94]	national	2009 – 2017	all ages	IPD overall	Proportion, Incidence, NA
	Canada (Ontario)	[95]	regional	2010 – 2011	all ages	IPD overall, bacteremic pneumonia, bacteremia	Severity, Proportion
	Spain	[96]	hospital	2011 – 2014	>18y	bacteremic pneumonia	Severity, Proportion
	Spain (Madrid)	[97]	regional	2012 – 2015	all ages	IPD overall	Proportion, AMR proportion
	UK	[98]	national	2010 – 2015	all ages	IPD overall, bacteremia, non-IPD	Proportion, AMR proportion
	Spain	[99]	hospital	2011 – 2015	>18y	bacteremic pneumonia	Proportion
	Portugal	[100]	national	2010 – 2015	<18y	complicated pneumonia	Proportion
	Portugal	[101]	national	2012 – 2015	<18y	IPD overall	Proportion
	Denmark	[102]	national	2010 – 2013	0-3mo	IPD overall	Proportion
	Denmark	[103]	national	2011 – 2014	all ages	IPD overall	Proportion, Incidence
	Norway	[104]	national	2011 – 2012	<5y	IPD overall	Proportion
	Taiwan	[105]	hospital	2012 – 2014	all ages	IPD overall	Proportion
	Taiwan	[106]	national	2007 – 2013	2mo-5y	IPD overall	Proportion
	US	[107]	national	2010 – 2017	>18y	all pneumonia, non-IPD	Proportion, AMR proportion
	Japan (Chiba)	[108]	regional	2016 – 2019	5-15y	all pneumonia	Proportion
	Cambodge	[109]	hospital	2012 – 2018	<5y	IPD overall	Proportion
	Japan	[110]	national	2011 – 2017	all ages	IPD overall	Proportion
	Japan	[111]	national	2016 – 2016	<15y	AOM	Proportion
EUR	Sweden (Southern)	[112]	regional	2016 – 2018	all ages	IPD + non-IPD, non-IPD, AOM	Proportion, AMR proportion
	Germany	[113]	national	2009 – 2018	all ages	IPD overall	Proportion
	Germany	[114]	national	2010 – 2014	all ages	IPD overall	Proportion

England	[115]	national	2002 – 2014	all ages	meningitis, IPD overall, comp.pneumonia, other IPD, bact.pneumo	Proportion, Severity
Spain (Tarragona)	[116]	regional	2012 – 2015	all ages	IPD overall	Proportion
S.Africa	[117]	national	2011 – 2012	all ages	IPD overall	Proportion, Incidence
Netherlands	[118]	national	2004 – 2012	all ages	IPD overall, bact.pneumonia, meningitis	Proportion, Severity
Netherlands	[119]	national	2008 – 2012	>18y	IPD overall	Proportion
UK	[120]	national	2013 – 2014	all ages	IPD overall	Incidence, Proportion
China	[121]	hospital	2011 – 2014	<5y	non-IPD, IPD overall	Proportion
Germany	[122]	national	2010 – 2014	5y:15y	IPD overall	Proportion
Germany	[123]	national	2014 – 2016	<16y	meningitis, IPD overall	Proportion
Canada (Ontario)	[124]	regional	2007 – 2017	all ages	IPD overall	Incidence, Proportion
South Korea	[125]	national	2011 – 2013	<18y	IPD overall	Proportion
Argentina	[126]	national	2013 – 2017	>18y	other IPD, IPD overall, bact. pneumonia, bacteremia, meningitis	Proportion
Israel	[127]	hospital	2010 – 2014	<2mo	AOM	Proportion

*All of the 7 PCV20nonPCV13 serotypes are reported.

Abbreviations: AFR = African Region; AMR (WHO region) = Americas; AMR (outcome) = antimicrobial resistance; Any disease = all *Streptococcus pneumoniae* clinical presentations; AOM = acute otitis media; EMR = Eastern Mediterranean Region; EUR = European Region; IPD = invasive pneumococcal disease; MULTI = multiple regions; SEAR = South-East Asia Region; UK = United Kingdom; US = United States of America; WPR = Western Pacific Region.

Table S4. PCV20nonPCV13 serotype-specific proportion (%) by clinical presentation: number of references (n), median (med.), and minimum and maximum (min–max).

	8		10A		11A		12F		15B/C		22F		33F	
	n	mean (min-max)	n	mean (min-max)	n	mean (min-max)	n	mean (min-max)	n	mean (min-max)	n	mean (min-max)	n	mean (min-max)
**Children**														
**No PCV**														
sterile site: IPD overall														
IPD, presentation unspecified[1]		nr	0	nr	1	6 (6–6)	0	nr	1	0 (0–0)	0	nr	0	nr
non-sterile site: non-IPD overall														
non-IPD, presentation specified:														
pneumococcal AOM		nr	0	nr	0	nr	0	nr	1	2.8 (2.8–2.8)	0	nr	0	nr
non-IPD, presentation unspecified		nr	0	nr	0	nr	0	nr	1	3.2 (3.2–3.2)	0	nr	0	nr
**PCV10, Initial period**														
sterile site: IPD overall														
IPD, presentation specified:														
other IPD[2]	1	2.3 (2.3–2.3)	0	nr	0	nr	1	2.3 (2.3–2.3)	1	7 (7–7)	1	2.3 (2.3–2.3)	0	nr
IPD, presentation unspecified	4	4.1 (0–10.5)	3	2.2 (1.8–11.1)	3	0.9 (0–1.6)	3	1.8 (0–3.6)	3	2.5 (0–2.8)	3	4.6 (2.5–5.6)	2	1.7 (1.4–2)
non-sterile site: non-IPD overall														
non-IPD, presentation specified:														
pneumococcal AOM	1	0.6 (0.6–0.6)	1	0.6 (0.6–0.6)	1	3 (3–3)	1	0 (0–0)	1	5.5 (5.5–5.5)	0	nr	1	0
**PCV10, Later period**														
sterile site: IPD overall														
IPD, presentation unspecified	1	0 (0–0)	1	13 (13–13)	1	0	1	0 (0–0)	1	8.7 (8.7–8.7)	1	4.3 (4.3–4.3)	1	21.7 (21.7–21.7)

non-sterile site: non-IPD overall														
non-IPD, presentation specified:														
pneumococcal AOM	0	nr	0	nr	1	7.1 (7.1–7.1)	0	nr	0	nr	0	nr	0	nr
**PCV13, Initial period**														
sterile site: IPD overall														
IPD, presentation specified:														
pneumococcal bacteremia	1	1.2 (1.2–1.2)	0	nr	0	nr	1	15.4 (15.4–15.4)	3	13.6 (4.3–23.1)	2	3.6 (2.2–5)	2	16.9 (8.7–25)
pneumococcal meningitis	0	nr	2	5.1 (3.6–6.5)	0	nr	2	10.7 (7.1–14.3)	3	5.8 (2.4–9.5)	3	1.2 (0–6.5)	3	3.9 (1.2–9.5)
bacteremic pneumococcal pneumonia	3	1.1 (0–2.5)	3	1.1 (0–2.1)	2	1.7 (1.3–2.1)	0	4.7 (4.3–5.1)	3	1.3 (0–6.5)	2	0 (0–2.5)	1	3.2 (3.2–3.2))
other IPD	0	nr	1	9.1 (9.1–9.1)	0	nr	0	nr	1	4.8 (4.8–4.8)	1	4.8 (4.8–4.8)	3	4.8 (4.8–4.8)
IPD, presentation unspecified	18	0.9 (0–11)	27	3.2 (0–11.1)	20	1.4 (0–6.8)	24	2 (0–24.2)	34	7 (0–33.3)	32	4.3 (0–13.2)	28	2.5 (0–11.1)
non-sterile site: non-IPD overall														
IPD, presentation specified:														
pneumococcal AOM	1	3.8 (3.8–3.8)	1	6.5 (6.5–6.5)	3	3.2 (0–18.8)	1	3.2 (3.2–3.2)	2	6.5 (0–12.9)	1	6.5 (6.5–6.5)	3	3.8 (3.2–12.5)
undifferentiated site: pneumo.disease														
pneumo. disease, presentation specified:														
all pneumococcal pneumonia	0	nr	2	5 (0–10.0)	0	nr	2	2.3 (0–4.5)	0	nr	0	nr	2	5.0 (0–10.0)
pneumo. disease, presentation unspecified	1	11.4 (11.4–11.4)	4	2.2 (0–4.3)	3	2.4 (1.9–3.7)	2	0 (0–0.3)	6	7.5 (5.3–13.4)	3	3.7 (2.4–7.7)	3	1 (0.7–2.1)
**PCV13, Later period**														
sterile site: IPD overall														
IPD, presentation specified:														
pneumococcal bacteremia	3	6.2 (0–11.2)	3	12 (4.7–12.5)	2	2.4 (0–4.7)	3	8.0 (4.7–13.1)	3	9.4 (0–9.6)	2	2.7 (0–5.4)	2	3.9 (0–7.7)
pneumococcal meningitis	3	6.2 (0–8.6)	4	5.8 (3.1–9.9)	1	0 (0–0)	4	4.8 (0–15.1)	3	9.4 (5.6–9.9)	3	6 (0–10.3)	3	5.8 (0–10.3)

bacteremic pneumococcal pneumonia	4	3 (0.5–5.6)	2	4.0 (0.5–7.4)	2	1.3 (0.9–1.6)	4	4.55 (0.5–12.2)	2	4.1 (0–8.1)	4	1.1 (0–6.3)	3	5.9 (0.5–6.6)
other IPD	2	6.2 (4.3–8.0)	2	5.7 (4.3–7.0)	1	8.7 (8.7–8.7)	3	10.7 (0–12.0)	3	8.7 (6–11.4)	2	6.4 (4.3–8.5)	3	7.0 (4.3–8.1)
IPD, presentation unspecified	17	2.4 (0–20)	19	5.8 (0–40)	13	1.4 (0–3.3)	20	6 (0–16.4)	18	5.2 (0–23.1)	17	4.6 (0–11.8)	17	3.3 (0–50)
non-sterile site: non-IPD overall														
non-IPD, presentation specified:														
pneumococcal AOM	1	1.8 (1.8–1.8)	2	6.9 (5.4–8.4)	2	7.3 (5.6–8.9)	2	0.5 (0–0.9)	2	10.6 (7.1–14)	2	3.7 (1.8–5.6)	2	2.3 (0.9–3.6)
non-IPD site unspecified	1	0 (0–0)	2	6.8 (4.8–8.7)	1	1 (1–1)	2	1.5 (0–2.9)	2	11.2 (4.8–17.5)	0	nr	1	0.3 (0.3–0.3)
undifferentiated site: pneumo. disease														
pneumo. disease, presentation specified														
all pneumococcal pneumonia	0	nr	1	1.6 (1.6–1.6)	1	10.9 (10.9–10.9)	0	nr	1	3.1 (3.1–3.1)	1	1.6 (1.6–1.6)	1	1.6 (1.6–1.6)
pneumo. disease, presentation unspecified	1	1 (1–1)	0	nr	2	10.9 (10.8–10.9)	0	nr	2	12.1 (11.3–12.8)	0	nr	2	1.2 (1–1.3)
**Adults**														
**No PCV**														
Sterile site: IPD overall														
IPD, presentation unspecified	2	6.0 (4.7–7.3)	2	1.1 (0.3–1.8)	2	0.5 (0–0.9)	2	0.9 (0–1.8)	2	0.2 (0–0.3)	1	0 (0–0)	2	0.5 (0–0.9)
**PCV10, Initial period**														
Sterile site: IPD overall														
IPD, presentation unspecified	2	10.1 (9.4–10.8)	2	1.7 (1.6–1.8)	2	1.7 (1.6–1.8)	1	2.1 (2.1–2.1)	1	0.7 (0.7–0.7)	2	5.4 (4.2–6.5)	2	2 (1.7–2.3)
**PCV13, Initial period**														
Sterile site: IPD overall														
IPD, presentation specified:														
bacteremic pneumococcal pneumonia	2	6.8 (5.3–8.3)	3	1.0 (0.7–3.1)	3	3.5 (1.0–10.8)	2	2.7 (1.2–4.2)	3	3.1 (1–4.2)	3	6.2 (3.6–7.5)	1	2.1 (2.1–2.1)
IPD, presentation unspecified	13	2.9 (0.1–22.2)	10	2.1 (0–10)	12	2.9 (0–7.5)	10	2.3 (1–4.3)	13	2.1 (0–8)	15	7.7 (0–13.1)	11	1.7 (0.8–3.1)

non-sterile site: non-IPD overall														
non-IPD site unspecified	5	0.8 (0.5–14.5)	6	1.3 (0.8–3.8)	3	5.8 (5.7–8.1)	2	1 (0.8–1.2)	5	5.3 (3.8–8)	3	3.5 (1.2–6.9)	3	1.2 (0–2.7)
undifferentiated site: pneumo. disease														
pneumo. disease, presentation specified:														
all pneumococcal pneumonia	6	1.8 (0–2.4)	5	0.6 (0–2.5)	7	3.4 (0.6–14.6)	5	1.0 (0–6.5)	5	0.6 (0–2.9)	6	4.6 (2.1–9.3)	5	1.3 (0.6–5.2)
pneumo. disease, presentation unspecified	2	3.3 (2.5–4)	2	3.4 (2.7–4)	2	3.8 (2.5–5)	1	1 (1–1)	2	3.4 (1.3–5.4)	1	3 (3–3)	0	nr
**PCV13, Later period**														
sterile site: IPD overall														
IPD, presentation specified:														
pneumococcal bacteremia	2	10.1 (6.9–13.2)	1	0 (0–0)	2	4.8 (3.6–5.9)	2	7.3 (6.6–7.9)	1	2 (2–2)	2	5.1 (2–8.2)	2	2.8 (2.0–3.6)
pneumococcal meningitis	2	8.2 (5.1–11.2)	2	3.4 (3.1–3.6)	2	3 (2–4)	2	9.1 (9–9.2)	2	2.1 (1–3.2)	2	5.6 (5.1–6.1)	1	2 (2–2)
bacteremic pneumococcal pneumonia	3	10.6 (5.2–15.6)	2	0.8 (0.7–0.9)	3	2.6 (1.7–3.8)	3	8.1 (3.5–8.8)	2	1.4 (1.2–1.5)	3	4.4 (4.1–8.4)	3	0.9 (0.7–3.9)
other IPD	2	5.1 (0–10.2)	2	3.1 (2–4.2)	1	7.8 (7.8–7.8)	2	8.2 (7.8–8.6)	1	5.9 (5.9–5.9)	2	4.8 (2–7.6)	2	2.1 (0–4.2)
IPD, presentation unspecified	35	13.6 (3.7–50)	15	2.8 (0–5.5)	18	3 (0–5.3)	29	6.5 (0–22.7)	18	1.5 (0–4.5)	25	7.4 (1.6–50)	20	2.6 (0.9–14.5)
non-sterile site: non-IPD overall														
non-IPD, presentation unspecified	4	2 (1.8–5.3)	4	1.8 (0–2.7)	1	0 (0–0)	2	0 (0–3.8)	4	2.1 (0–2.7)	1	1.9 (1.9–1.9)	3	0.5 (0.3–1.9)
undifferentiated site: pneumo. disease														
pneumo. disease, presentation specified:														
all pneumococcal pneumonia	9	20.2 (0.9–50.5)	9	3.2 (0–5.5)	10	5.1 (0.5–16.8)	9	5.8 (0–21.9)	4	1 (0–1.9)	10	4.8 (0.8–13.1)	9	2.7 (0.9–12.7)
pneumo. disease, presentation unspecified	1	2.1 (2.1–2.1)	0	nr	1	11 (11–11)	0	nr	1	6.8 (6.8–6.8)	0	nr	1	0.6 (0.6–0.6)
**All ages**														
**No PCV**														
undifferentiated site: pneumo. disease														

pneumo. disease, presentation unspecified	0	nr	1	0.7 (0.7–0.7)	1	2.2 (2.2–2.2)	0	nr	1	1.1 (1.1–1.1)	0	nr	1	0.4 (0.4–0.4)
**PCV10, Initial period**														
sterile site: IPD overall														
IPD, presentation unspecified	1	3.7 (3.7–3.7)	1	1.6 (1.6–1.6)	1	1.9 (1.9–1.9)	1	1.4 (1.4–1.4)	1	0.7 (0.7–0.7)	1	0.2 (0.2–0.2)	1	0.5 (0.5–0.5)
**PCV13, Initial period**														
sterile site: IPD overall														
IPD, presentation specified:														
pneumococcal bacteremia	3	9.6 (7.6–11.8)	0	nr	1	3.2 (3.2–3.2)	2	3.9 (3–4.8)	0	nr	3	7.8 (4.4–10.9)	2	5.4 (5.2–5.6)
pneumococcal meningitis	4	1.6 (0.8–2.8)	4	4.5 (2.5–5.7)	4	2.6 (1.8–3.4)	4	11.1 (5.8–13.4)	4	4.3 (2.9–6.3)	3	4 (3.9–5.2)	3	2.2 (1.9–2.3)
IPD, presentation unspecified	14	5.4 (0–15.2)	11	1.5 (0–3.9)	13	2.5 (0–5.6)	11	2.8 (1.1–6.8)	11	2.6 (0.6–16.7)	16	7.2 (0–11.5)	13	2.8 (0.6–4.6)
non-sterile site: non-IPD overall														
non-IPD, presentation unspecified	0	nr	0	nr	1	5.6 (5.6–5.6)	1	0.2 (0.2–0.2)	1	7 (7–7)	1	4.4 (4.4–4.4)	0	nr
undifferentiated site: pneumo. disease														
pneumo. disease, presentation unspecified	1	3.2 (3.2–3.2)	2	3.1 (0.8–5.4)	2	5.15 (1.6–8.7)	0	nr	2	1.75 (1.1–2.4)	1	1.6 (1.6–1.6)	0	nr
**PCV13, Later period**														
sterile site: IPD overall														
IPD, presentation specified:														
pneumococcal bacteremia	4	15.4 (0–17.4)	3	3.2 (0–4.3)	2	1.5 (0–3.0)	4	6.0 (0–11.6)	1	0 (0–0)	4	6.8 (0–8.9)	3	3.4 (0–3.8)
pneumococcal meningitis	4	9.6 (3.2–12.8)	4	6.2 (5.8–8.3)	2	1.6 (0–3.2)	3	6.8 (0–14)	2	10.5 (4.3–16.7)	4	7.6 (5.4–25)	4	2.9 (0–5.8)
bacteremic pneumococcal pneumonia	2	18 (14–22)	2	1.1 (0–2.1)	1	7 (7–7)	2	6.7 (1.8–11.6)	1	3.5 (3.5–3.5)	2	5.4 (3.5–7.3)	2	4.5 (3.6–5.3)
other IPD	1	11.6 (11.6–11.6)	1	4.8 (4.8–4.8)	0	nr	1	13.5 (13.5–13.5)	0	nr	1	6.6 (6.6–6.6)	1	4.0 (4.0–4.0)
IPD, presentation unspecified	11	15.3 (1.6–31.6)	8	2.5 (0.8–4)	7	2.2 (0–5.6)	10	7 (1.4–13.1)	6	1.8 (0.7–2.8)	10	6.9 (2–8.4)	8	3.5 (0–4.4)
non-sterile site: non-IPD overall														

non-IPD, presentation specified:														
pneumococcal AOM	1	1.4 (1.4–1.4)	0	nr	1	2.8 (2.8–2.8)	0	nr	1	8.5 (8.5–8.5)	0	nr	0	nr
non-IPD, presentation unspecified	1	2.1 (2.1–2.1)	0	nr	1	10.7 (10.7–10.7)	0	nr	1	7.6 (7.6–7.6)	0	nr	1	0.9 (0.9–0.9)

AOM=acute otitis media; IPD=invasive pneumococcal disease; nr=not reported

[1] Refers to cases where the precise clinical presentation was not reported.

[2] Infections of other normally sterile sites (joint fluid, pericardial fluid, peritoneal fluid, infected bone, among others).

Table S5. Meta-analysis of serotype-specific proportion * of IPD isolates from children and adults.

Children	Serotype	N (Studies)	Proportion (95% CI)
**PCV13 NIP, initial period (<3 years)**			
	**All PCV13**	26	50.3 (41.3–59.3)
	**PCV20nonPCV13**		
	**8**	22	2.1 (1.3–3.3)
	**10A**	24	3.5 (2.8–4.4)
	**11A**	20	1.9 (1.3–2.9)
	**12F**	24	4.4 (2.6–7.6)
	**15B/C**	31	7.2 (5.6–9.1)
	**22F**	25	3.6 (2.5–5.3)
	**33F**	23	3.2 (2.3–4.6)
**PCV13 NIP, later period (≥3 years)**			
	**All PCV13**	17	44.5 (30.6–59.3)
	**PCV20nonPCV13**		
	**8**	19	3.8 (2.5–5.8)
	**10A**	18	5.9 (4.5–7.7)
	**11A**	15	1.9 (1.6–2.3)
	**12F**	20	4.9 (3.2–7.4)
	**15B/C**	18	6.2 (4.4–8.7)
	**22F**	20	4.2 (2.7–6.3)
	**33F**	18	4.4 (3.0–6.4)
**Adults**	**Serotype**	**N (Studies)**	**Proportion (95% CI)**
**PCV13 NIP, initial period (<3 years)**			
	**All PCV13**	13	49.5 (45.0–57.0)
	**PCV20nonPCV13**		
	**8**	10	4.1 (1.6–10.4)
	**10A**	8	1.8 (1.0–3.5)
	**11A**	10	3.7 (2.4–5.6)
	**12F**	7	2.4 (1.5–4.0)
	**15B/C**	11	2.5 (1.7–3.6)
	**22F**	13	7.7 (5.9–9.9)
	**33F**	7	1.8 (1.2–2.7)
**PCV13 NIP, later period (≥3 years)**			
	**All PCV13**	9	27.3 (19.6–36.7)
	**PCV20nonPCV13**		
	**8**	13	10.2 (7.6–13.4)
	**10A**	13	2.4 (1.9–3.0)
	**11A**	13	2.7 (2.4–3.1)
	**12F**	13	6.2 (4.7–8.1)
	**15B/C**	11	1.5 (1.2–2.0)
	**22F**	13	6.7 (5.7–7.8)
	**33F**	12	2.5 (1.8–3.4)

* Calculated with a binomial random-effects meta-analytic model for each serotype-specific proportion independently. Consequently, the sum of all PCV20nonPCV13 serotypes may exceed 100%.

Table S6: Sensitivity analysis restricted to studies without moderate or serious risk of bias: Subset meta-analysis of serotype-specific proportion* of IPD isolates from children and adults.

Children	Serotype	N(studies)	Proportion (95%CI)
	**PCV13 NIP, Initial period (<3 years)**		
	**All PCV13**	11	56.3 (43.7 – 69.4)
	**PCV20nonPCV13**		
	**8**	11	2.3 (1.3 – 4.2)
	**10A**	13	2.6 (2.1 – 3.4)
	**11A**	11	2.0 (1.1 – 3.5)
	**12F**	11	3.2 (0.9 – 10.6)
	**15B/C**	16	6.6 (4.5 – 9.4)
	**22F**	14	3.1 (1.6 – 6.1)
	**33F**	11	2.5 (1.3 – 4.6)
	**PCV13 NIP, Later period (≥3 years)**		
	**All PCV13**	10	46.3 (33.0 – 60.1)
	**PCV20nonPCV13**		
	**8**	10	3.6 (1.5 – 8.1)
	**10A**	11	4.4 (3.1 – 6.2)
	**11A**	10	2.2 (1.9 – 2.6)
	**12F**	11	4.0 (2.2 – 6.9)
	**15B/C**	11	5.6 (2.9 – 10.5)
	**22F**	10	3.1 (1.5 – 6.3)
	**33F**	11	3.2 (1.7 – 6.2)

Adults	Serotype	N(studies)	Proportion (95%CI)
	**PCV13 NIP, Initial period (<3years)**		
	**All PCV13**	7	50.9 (38.8 – 63)
	**PCV20nonPCV13**		
	**8**	5	6.1 (1.5 – 21.3)
	**10A**	2	1.8 (0 – 99.0)
	**11A**	4	3.6 (1.1 – 10.9)
	**12F**	2	1.6 (0.5 – 5.0)
	**15B/C**	5	2.9 (1.3 – 6.5)
	**22F**	6	10.8 (8.6 – 13.4)
	**33F**	3	2.5 (1.2 – 5.0)
	**PCV13 NIP, Later period (≥3years)**		
	**All PCV13**	6	34.4 (22.6 – 48.5)
	**PCV20nonPCV13**		
	**8**	8	9.1 (5.9 – 13.9)
	**10A**	8	2.2 (1.5 – 3.1)
	**11A**	8	2.7 (2.5 – 3.0)
	**12F**	8	6.6 (5.1 – 8.5)
	**15B/C**	6	1.8 (1.6 – 2.1)
	**22F**	8	6.6 (5.1 – 8.4)
	**33F**	7	2.4 (1.3 – 4.5)

Table S7. Incidence rate of PCV20nonPCV13 serotypes per 100,000 person-years (if not stated otherwise).

Sampling site	Clinical presentation	Region	Country	Study period	Age	Reference	8	10A	11A	12F	15B/C	22F	33F
**Children**													
Sterile site: IPD	IPD, presentation specified												
	Pneumococcal meningitis	EUR	Israel	2008–2016	<5y	[10]	0.1	0.1	nr	0.4	0.1	0.1	0.1
	Bacteremic pneumococcal pneumonia	EUR	Israel	2008–2016	<5y	[10]	0.1	<0.1	nr	0.8	0.1	0.1	0.3
	Other IPD[1]	EUR	Israel	2008–2016	<5y	[10]	<0.1	0.3	nr	1.8	0.6	0.1	0.4
	IPD presentation unspecified[2]	AFR	South Africa	2011–2012	<16y	[117]	0.4	0.1	nr	0.7	0.2	0.2	nr
		AMR	US (Alaska)	2010–2013	<5y	[12]	0.0	1.0	0.5	2.0	3.5	2.0	1.5
		EUR	Austria	2013–2016	<5y	[94]	nr	0.2[3]	nr	nr	0.2[3]	0.1[3]	nr
		EUR	Denmark	2011–2014	<5y	[103]	0.2	nr	0.1	0.6	nr	0.4	0.5
		EUR	Israel	2010–2013	<5y	[9]	nr	nr	nr	3.7	1.1	nr	0.8
		WPR	Australia	2014–2014	<5y	[46]	0.1	0.7	0.3	0.1	1.8	0.4	0.5
Non-sterile site: non-IPD	Non-IPD, presentation specified												

	Pneumococcal AOM	EUR	Israel (South)	2010–2013	<2y	[9]	nr	nr	0.1[4]	nr	0.3[4]	nr	nr
**Adults**													
Sterile site: IPD	IPD, presentation specified	EUR	Austria	2013–2016	>50y	[94]	0.3[3]	nr	0.2[3]	nr	nr	0.3[3]	nr
		EUR	Denmark	2011–2014	≥65y	[103]	4.8	nr	1.3	2.6	nr	3.8	1.7
		EUR	Spain (Catalonia)	2014–2016	>65y	[22]	2.8	0.8	0.9	2.4	0.3	1.8	0.6
		EUR	UK (Nottingham)	2013–2018	>16y	[90]	2.0	nr	nr	1.1	nr	0.8	nr
		WPR	Australia	2014–2014	≥65y	[46]	0.2	0.1	0.6	0.1	0.5	1.6	0.5
Undifferentiated site	All pneumococcal pneumonia	EUR	UK (Nottingham)	2013–2018	>16y	[90]	6.9	nr	nr	2.1	nr	1.3	nr
**All ages**													
Sterile site: IPD	IPD, presentation unspecified[2]	AMR	Canada (Ontario)	2011–2017	all	[124]	nr	nr	nr	nr	nr	0.8	nr
		AMR	US (Alaska)	2010–2013	all	[12]	0.8	0.4	0,4	0.7	0.5	1.4	0.4
		EUR	France	2012–2012	all	[62]	nr	nr	nr	1.7	1.5	1.0	nr

Abbreviations: AFR = Africa; AMR = Americas; EUR = Europe; WPR = Western Pacific; nr = not reported; other IPD = any IPD excluding meningitis and bacteremic pneumonia.

[1] Infections of other normally sterile sites (joint fluid, pericardial fluid, peritoneal fluid, infected bone, among others).

[2] Refers to cases where the precise clinical presentation was not reported.

[3] per 100,000 person-months.

[4] per 1,000 population.

Table S8. Proportion (%) of PCV20nonPCV13 serotypes non-susceptible to penicillin or a macrolide or that were multi-drug resistant (MDR)

Age group	Sampling site and clinical pres.	Antibiotic class	Reference	8	10A	11A	12F	15B/C	22F	33F
Children	Sterile site (IPD)									
	IPD presentation unspecified	Penicillin	[44]	0	0	40	0	0	0	nr
	Non-sterile site (Non-IPD)									
	Non-IPD presentation unspecified	Penicillin	[81]	nr	nr	0	nr	0	nr	nr
	Non-IPD presentation unspecified	Macrolide	[81]	nr	nr	60	nr	33	nr	nr
	Undifferentiated site									
	Pneumo. disease presentation unsp.	Penicillin	[80]	nr	nr	nr	nr	nr	0	nr
Adults	Non-sterile site (Non-IPD)									
	Non-IPD presentation unspecified	Penicillin	[31]	nr	0	nr	nr	nr	nr	nr
	Non-IPD presentation unspecified	Macrolide	[31]	nr	5	nr	nr	nr	nr	nr
	Non-IPD presentation unspecified	Penicillin	[73]	nr	1	nr	nr	21	nr	nr
	Non-IPD presentation unspecified	Macrolide	[73]	nr	10	nr	nr	57	nr	nr
	Non-IPD presentation unspecified	Penicillin	[107]	nr	nr	2	nr	nr	0	nr
	Non-IPD presentation unspecified	Macrolide	[107]	nr	nr	30	nr	nr	17	nr
	Non-IPD presentation unspecified	MDR	[107]	nr	nr	2	nr	nr	0	nr
	Undifferentiated site									
	Pneumo. disease presentation unsp.	Penicillin	[6]	0	0	0	0	0	0	0
	Pneumo. disease presentation unsp.	Penicillin	[107]	nr	nr	0	nr	nr	0	nr
	Pneumo. disease presentation unsp.	Macrolide	[107]	nr	nr	41	nr	nr	26	nr
	Pneumo. disease presentation unsp.	MDR	[107]	nr	nr	3	nr	nr	1	nr
All ages	Sterile site (IPD)									
	IPD presentation unspecified	Penicillin	[24]	0	0	25	nr	nr	nr	0
	IPD presentation unspecified	Macrolide	[24]	11	100	0	nr	nr	nr	100
	IPD presentation unspecified	Penicillin	[36]	nr	nr	nr	nr	nr	0	0
	IPD presentation unspecified	Macrolide	[36]	nr	nr	nr	nr	nr	26	79

IPD presentation unspecified	MDR	[36]	nr	nr	nr	nr	nr	1	9
IPD presentation unspecified	Penicillin	[74]	nr	nr	0	nr	nr	nr	nr
IPD presentation unspecified	Macrolide	[74]	2	2	6	nr	3	nr	9
IPD presentation unspecified	Penicillin	[93]	nr	nr	0	nr	8	nr	nr
IPD presentation unspecified	Penicillin	[97]	48	nr	nr	2	2	nr	nr
Non-sterile site (Non-IPD)									
Non-IPD presentation unspecified	Penicillin	[93]	nr	nr	10	nr	39	nr	nr
Non-IPD presentation unspecified	MDR	[98]	4	0	4	nr	0	0	nr
Undifferentiated site									
Pneumo. disease presentation unsp.	Penicillin	[51]	nr	0	nr	nr	22	nr	nr
Pneumo. disease presentation unsp.	Macrolide	[51]	nr	73	nr	nr	100	nr	nr
Pneumo. disease presentation unsp.	Penicillin	[53]	nr	0	33	nr	67	nr	0
Pneumo. disease presentation unsp.	Macrolide	[53]	nr	50	33	nr	33	nr	0
Pneumo. disease presentation unsp.	Penicillin	[112]	nr	nr	5	nr	10	nr	nr
Pneumo. disease presentation unsp.	Macrolide	[112]	nr	nr	3	nr	7	nr	38

Abbreviations: IPD = invasive pneumococcal disease; MDR = multi-drug resistant; nr = not reported.

References

1. Aguiar, S.I.; Brito, M.J.; Horacio, A.N.; Lopes, J.P.; Ramirez, M.; Melo-Cristino, J.; Portuguese Group for the Study of Streptococcal, I.; Portuguese Study Group of Invasive Pneumococcal Disease of the Paediatric Infectious Disease, S. Decreasing incidence and changes in serotype distribution of invasive pneumococcal disease in persons aged under 18 years since introduction of 10-valent and 13-valent conjugate vaccines in Portugal, July 2008 to June 2012. *Euro Surveill.* **2014**, *19*, 20750.
2. Alari, A.; Chaussade, H.; Domenech De Celles, M.; Le Fouler, L.; Varon, E.; Opatowski, L.; Guillemot, D.; Watier, L. Impact of pneumococcal conjugate vaccines on pneumococcal meningitis cases in France between 2001 and 2014: a time series analysis. *BMC Med.* **2016**, *14*, 211, doi:10.1186/s12916-016-0755-7.
3. Al-Jardani, A.; Al Rashdi, A.; Al Jaaidi, A.; Al Bulushi, M.; Al Mahrouqi, S.; Al-Abri, S.; Al-Maani, A.; Kumar, R. Serotype distribution and antibiotic resistance among invasive *Streptococcus pneumoniae* from Oman post 13-valent vaccine introduction. *Int. J. Infect. Dis.* **2019**, *85*, 135-140, doi:10.1016/j.ijid.2019.05.027.
4. Amin-Chowdhury, Z.; Collins, S.; Sheppard, C.; Litt, D.; Fry, N.K.; Andrews, N.; Ladhani, S.N. Characteristics of invasive pneumococcal disease caused by emerging serotypes after the introduction of the 13-valent pneumococcal conjugate vaccine in England: a prospective observational cohort study, 2014-2018. *Clin. Infect. Dis.* **2020**, *71*, e235-e243, doi:10.1093/cid/ciaa043.
5. Angoulvant, F.; Levy, C.; Grimprel, E.; Varon, E.; Lorrot, M.; Biscardi, S.; Minodier, P.; Dommergues, M.A.; Hees, L.; Gillet, Y.; et al. Early impact of 13-valent pneumococcal conjugate vaccine on community-acquired pneumonia in children. *Clin. Infect. Dis.* **2014**, *58*, 918-924.
6. Bedos, J.P.; Varon, E.; Porcher, R.; Asfar, P.; Le Tulzo, Y.; Megarbane, B.; Mathonnet, A.; Dugard, A.; Veinstein, A.; Ouchenir, K.; et al. Host-pathogen interactions and prognosis of critically ill immunocompetent patients with pneumococcal pneumonia: the nationwide prospective observational STREPTOGENE study. *Intensive Care Med.* **2018**, *44*, 2162-2173, doi:10.1007/s00134-018-5444-x.
7. Benfield, T.; Skovgaard, M.; Schonheyder, H.C.; Knudsen, J.D.; Bangsborg, J.; Ostergaard, C.; Slotved, H.C.; Konradsen, H.B.; Thomsen, R.W.; Lambertsen, L. Serotype distribution in non-bacteremic pneumococcal pneumonia: association with disease severity and implications for pneumococcal conjugate vaccines. *PLoS One* **2013**, *8*, e72743.
8. Ben-Shimol, S.; Givon-Lavi, N.; Leibovitz, E.; Raiz, S.; Greenberg, D.; Dagan, R. Near-elimination of otitis media caused by 13-valent pneumococcal conjugate vaccine (PCV) serotypes in southern Israel shortly after sequential introduction of 7-valent/13-valent PCV. *Clin. Infect. Dis.* **2014**, *59*, 1724-1732.
9. Ben-Shimol, S.; Greenberg, D.; Givon-Lavi, N.; Schlesinger, Y.; Somekh, E.; Aviner, S.; Miron, D.; Dagan, R. Early impact of sequential introduction of 7-valent and 13-valent pneumococcal conjugate vaccine on IPD in Israeli children <5 years: an active prospective nationwide surveillance. *Vaccine* **2014**, *32*, 3452-3459.
10. Ben-Shimol, S.; Givon-Lavi, N.; Grisaru-Soen, G.; Megged, O.; Greenberg, D.; Dagan, R.; Israel, B.; Meningitis Active Surveillance, G. Comparative incidence dynamics and serotypes of meningitis, bacteremic pneumonia and other-IPD in young children in the PCV era: Insights from Israeli surveillance studies. *Vaccine* **2018**, *36*, 5477-5484, doi:10.1016/j.vaccine.2017.05.059.
11. Bewick, T.; Sheppard, C.; Greenwood, S.; Slack, M.; Trotter, C.; George, R.; Lim, W.S. Serotype prevalence in adults hospitalised with pneumococcal non-invasive community-acquired pneumonia. *Thorax* **2012**, *67*, 540-545, doi:10.1136/thoraxjnl-2011-201092.
12. Bruce, M.G.; Singleton, R.; Bulkow, L.; Rudolph, K.; Zulz, T.; Gounder, P.; Hurlburt, D.; Bruden, D.; Hennessy, T. Impact of the 13-valent pneumococcal conjugate vaccine (pcv13) on invasive pneumococcal disease and carriage in Alaska. *Vaccine* **2015**, *33*, 4813-4819, doi:10.1016/j.vaccine.2015.07.080.

13. Cabaj, J.L.; Nettel-Aguirre, A.; MacDonald, J.; Vanderkooi, O.G.; Kellner, J.D. Influence of childhood pneumococcal conjugate vaccines on invasive pneumococcal disease in adults with underlying comorbidities in Calgary, Alberta (2000-2013). *Clin. Infect. Dis.* **2016**, *62*, 1521-1526.
14. Ceyhan, M.; Ozsurekci, Y.; Gurler, N.; Oksuz, L.; Aydemir, S.; Ozkan, S.; Yuksekkaya, S.; Keser Emiroglu, M.; Gultekin, M.; Yaman, A.; et al. Serotype distribution of Streptococcus pneumoniae in children with invasive diseases in Turkey: 2008-2014. *Hum. Vaccin. Immunother.* **2016**, *12*, 308-313, doi:10.1080/21645515.2015.1078952.
15. Ceyhan, M.; Aykac, K.; Gurler, N.; Ozsurekci, Y.; Oksuz, L.; Altay Akisoglu, O.; Oz, F.N.; Emiroglu, M.; TurkDagi, H.; Yaman, A.; et al. Serotype distribution of Streptococcus pneumonia in children with invasive disease in Turkey: 2015-2018. *Hum. Vaccin. Immunother.* **2020**, *16*, 2773-2778, doi:10.1080/21645515.2020.1747931.
16. Chen, H.H.; Li, H.C.; Su, L.H.; Chiu, C.H. Fluoroquinolone-nonsusceptible *Streptococcus pneumoniae* isolates from a medical center in the pneumococcal conjugate vaccine era. *J. Microbiol. Immunol. Infect.* **2017**, *50*, 839-845, doi:10.1016/j.jmii.2016.05.003.
17. Chi, H.C.; Hsieh, Y.C.; Tsai, M.H.; Lee, C.H.; Kuo, K.C.; Huang, C.T.; Huang, Y.C. Impact of pneumococcal conjugate vaccine in children on the serotypic epidemiology of adult invasive pneumococcal diseases in Taiwan. *J. Microbiol. Immunol. Infect.* **2018**, *51*, 332-336, doi:10.1016/j.jmii.2016.08.009.
18. Cho, E.Y.; Choi, E.H.; Kang, J.H.; Kim, K.H.; Kim, D.S.; Kim, Y.J.; Ahn, Y.M.; Eun, B.W.; Oh, S.H.; Cha, S.H.; et al. Early changes in the serotype distribution of invasive pneumococcal isolates from children after the introduction of extended-valent pneumococcal conjugate vaccines in Korea, 2011-2013. *J. Korean Med. Sci.* **2016**, *31*, 1082-1088, doi:10.3346/jkms.2016.31.7.1082.
19. Cho, Y.C.; Chiu, N.C.; Lu, C.Y.; Huang, D.T.; Huang, F.Y.; Chang, L.Y.; Huang, L.M.; Chi, H. Redistribution of *Streptococcus pneumoniae* serotypes after nationwide 13-valent pneumococcal conjugate vaccine program in children in northern Taiwan. *Pediatr. Infect. Dis. J.* **2017**, *36*, e334-e340, doi:10.1097/inf.0000000000001664.
20. Choi, M.J.; Song, J.Y.; Cheong, H.J.; Jeon, J.H.; Kang, S.H.; Jung, E.J.; Noh, J.Y.; Kim, W.J. Clinical usefulness of pneumococcal urinary antigen test, stratified by disease severity and serotypes. *J Infect Chemother* **2015**, *21*, 672-679, doi:10.1016/j.jiac.2015.06.003.
21. Choi, M.J.; Noh, J.Y.; Cheong, H.J.; Kim, W.J.; Kim, M.J.; Jang, Y.S.; Lee, S.N.; Choi, E.H.; Lee, H.J.; Song, J.Y. Spread of ceftriaxone non-susceptible pneumococci in South Korea: Long-term care facilities as a potential reservoir. *PLoS One* **2019**, *14*, e0210520, doi:10.1371/journal.pone.0210520.
22. Ciruela, P.; Broner, S.; Izquierdo, C.; Pallarés, R.; Muñoz-Almagro, C.; Hernández, S.; Grau, I.; Domínguez, A.; Jané, M. Indirect effects of paediatric conjugate vaccines on invasive pneumococcal disease in older adults. *Int. J. Infect. Dis.* **2019**, *86*, 122-130, doi:10.1016/j.ijid.2019.06.030.
23. Cohen, C.; von Mollendorf, C.; de Gouveia, L.; Lengana, S.; Meiring, S.; Quan, V.; Nguweneza, A.; Moore, D.P.; Reubenson, G.; Moshe, M.; et al. Effectiveness of the 13-valent pneumococcal conjugate vaccine against invasive pneumococcal disease in South African children: a case-control study. *Lancet Glob Health* **2017**, *5*, e359-e369, doi:10.1016/S2214-109X(17)30043-8.
24. Correa, M.; Onieva-García, M.Á.; López, I.; Montiel, N. [Invasive neumococcal disease in Costa del Sol Hospital: replacement by non-vaccinable serotypes]. *Rev. Esp. Salud Publica* **2018**, *92*, e201806034.
25. Danis, K.; Varon, E.; Lepoutre, A.; Janssen, C.; Forestier, E.; Epaulard, O.; N'Guyen, Y.; Labrunie, A.; Lanotte, P.; Gravet, A.; et al. Factors Associated With Severe Nonmeningitis Invasive Pneumococcal Disease in Adults in France. *Open Forum Infect Dis* **2019**, *6*, ofz510, doi:10.1093/ofid/ofz510.
26. Demczuk, W.H.; Martin, I.; Griffith, A.; Lefebvre, B.; McGeer, A.; Lovgren, M.; Tyrrell, G.J.; Desai, S.; Sherrard, L.; Adam, H.; et al. Serotype distribution of invasive *Streptococcus pneumoniae* in Canada after the introduction of the 13-valent pneumococcal conjugate vaccine, 2010-2012. *Can. J. Microbiol.* **2013**, *59*, 778-788.

27. Desmet, S.; Lagrou, K.; Wyndham-Thomas, C.; Braeye, T.; Verhaegen, J.; Maes, P.; Fieuws, S.; Peetermans, W.E.; Blumental, S. Dynamic changes in paediatric invasive pneumococcal disease after sequential switches of conjugate vaccine in Belgium: a national retrospective observational study. *Lancet Infect. Dis.* **2021**, *21*, 127-136, doi:10.1016/S1473-3099(20)30173-0.
28. Djennad, A.; Ramsay, M.E.; Pebody, R.; Fry, N.K.; Sheppard, C.; Ladhani, S.N.; Andrews, N.J. Effectiveness of 23-valent polysaccharide pneumococcal vaccine and changes in invasive pneumococcal disease incidence from 2000 to 2017 in those aged 65 and over in England and Wales. *EClinicalMedicine* **2018**, *6*, 42-50, doi:10.1016/j.eclinm.2018.12.007.
29. Domingues, C.M.; Verani, J.R.; Montenegro Renoiner, E.I.; de Cunto Brandileone, M.C.; Flannery, B.; de Oliveira, L.H.; Santos, J.B.; de Moraes, J.C.; Brazilian Pneumococcal Conjugate Vaccine Effectiveness Study, G. Effectiveness of ten-valent pneumococcal conjugate vaccine against invasive pneumococcal disease in Brazil: a matched case-control study. *Lancet Respir Med* **2014**, *2*, 464-471.
30. Escribano Montaner, A.; Garcia de Lomas, J.; Villa Asensi, J.R.; Asensio de la Cruz, O.; de la Serna Blazquez, O.; Santiago Burruchaga, M.; Mondejar Lopez, P.; Torrent Vernetta, A.; Feng, Y.; Van Dyke, M.K.; et al. Bacteria from bronchoalveolar lavage fluid from children with suspected chronic lower respiratory tract infection: results from a multi-center, cross-sectional study in Spain. *Eur. J. Pediatr.* **2018**, *177*, 181-192, doi:10.1007/s00431-017-3044-3.
31. Fenoll, A.; Aguilar, L.; Gimenez, M.J.; Vicioso, M.D.; Robledo, O.; Granizo, J.J.; Coronel, P. Variations in serotypes and susceptibility of adult non-invasive *Streptococcus pneumoniae* isolates between the periods before (May 2000-May 2001) and 10 years after (May 2010-May 2011) introduction of conjugate vaccines for child immunisation in Spain. *Int. J. Antimicrob. Agents* **2012**, *40*, 18-23, doi:10.1016/j.ijantimicag.2012.03.001.
32. Fu, J.; Li, L.; Liang, Z.; Xu, S.; Lin, N.; Qin, P.; Ye, X.; McGrath, E. Etiology of acute otitis media and phenotypic-molecular characterization of *Streptococcus pneumoniae* isolated from children in Liuzhou, China. *BMC Infect. Dis.* **2019**, *19*, 168, doi:10.1186/s12879-019-3795-8.
33. Galanis, I.; Lindstrand, A.; Darenberg, J.; Browall, S.; Nannapaneni, P.; Sjostrom, K.; Morfeldt, E.; Naucler, P.; Blennow, M.; Ortqvist, A.; et al. Effects of PCV7 and PCV13 on invasive pneumococcal disease and carriage in Stockholm, Sweden. *Eur. Respir. J.* **2016**, *47*, 1208-1218.
34. Gentile, A.; Bakir, J.; Firpo, V.; Casanueva, E.V.; Ensinck, G.; Lopez Papucci, S.; Lucion, M.F.; Abate, H.; Cancellara, A.; Molina, F.; et al. PCV13 vaccination impact: A multicenter study of pneumonia in 10 pediatric hospitals in Argentina. *PLoS One* **2018**, *13*, e0199989, doi:10.1371/journal.pone.0199989.
35. Goettler, D.; Streng, A.; Kemmling, D.; Schoen, C.; von Kries, R.; Rose, M.A.; van der Linden, M.; Liese, J.G. Increase in Streptococcus pneumoniae serotype 3 associated parapneumonic pleural effusion/empyema after the introduction of PCV13 in Germany. *Vaccine* **2020**, *38*, 570-577, doi:10.1016/j.vaccine.2019.10.056.
36. Golden, A.R.; Adam, H.J.; Zhanel, G.G. Invasive Streptococcus pneumoniae in Canada, 2011-2014: Characterization of new candidate 15-valent pneumococcal conjugate vaccine serotypes 22F and 33F. *Vaccine* **2016**, *34*, 2527-2530, doi:10.1016/j.vaccine.2016.03.058.
37. Guevara, M.; Ezpeleta, C.; Gil-Setas, A.; Torroba, L.; Beristain, X.; Aguinaga, A.; Garcia-Irure, J.J.; Navascues, A.; Garcia-Cenoz, M.; Castilla, J.; et al. Reduced incidence of invasive pneumococcal disease after introduction of the 13-valent conjugate vaccine in Navarre, Spain, 2001-2013. *Vaccine* **2014**, *32*, 2553-2562, doi:10.1016/j.vaccine.2014.03.054.
38. Hare, K.M.; Smith-Vaughan, H.C.; Chang, A.B.; Pizzutto, S.; Petsky, H.L.; McCallum, G.B.; Leach, A.J. Propensity of pneumococcal carriage serotypes to infect the lower airways of children with chronic endobronchial infections. *Vaccine* **2017**, *35*, 747-756, doi:10.1016/j.vaccine.2016.12.059.
39. Hernandez, S.; Munoz-Almagro, C.; Ciruela, P.; Soldevila, N.; Izquierdo, C.; Codina, M.G.; Diaz, A.; Moraga-Llop, F.; Garcia-Garcia, J.J.; Dominguez, A. Invasive Pneumococcal Disease and Influenza Activity in a Pediatric Population: Impact of PCV13 Vaccination in

Pandemic and Nonpandemic Influenza Periods. *J. Clin. Microbiol.* **2019**, *57*, e00363-00319, doi:10.1128/JCM.00363-19.
40. Horacio, A.N.; Silva-Costa, C.; Lopes, E.; Ramirez, M.; Melo-Cristino, J.; Portuguese Group for the Study of Streptococcal, I. Conjugate vaccine serotypes persist as major causes of non-invasive pneumococcal pneumonia in Portugal despite declines in serotypes 3 and 19A (2012-2015). *PLoS One* **2018**, *13*, e0206912, doi:10.1371/journal.pone.0206912.
41. Houri, H.; Tabatabaei, S.R.; Saee, Y.; Fallah, F.; Rahbar, M.; Karimi, A. Distribution of capsular types and drug resistance patterns of invasive pediatric Streptococcus pneumoniae isolates in Teheran, Iran. *Int. J. Infect. Dis.* **2017**, *57*, 21-26, doi:10.1016/j.ijid.2017.01.020.
42. Imohl, M.; Moller, J.; Reinert, R.R.; Perniciaro, S.; van der Linden, M.; Aktas, O. Pneumococcal meningitis and vaccine effects in the era of conjugate vaccination: results of 20 years of nationwide surveillance in Germany. *BMC Infect. Dis.* **2015**, *15*, 61, doi:10.1186/s12879-015-0787-1.
43. Isturiz, R.E.; Ramirez, J.; Self, W.H.; Grijalva, C.G.; Counselman, F.L.; Volturo, G.; Ostrosky-Zeichner, L.; Peyrani, P.; Wunderink, R.G.; Sherwin, R.; et al. Pneumococcal epidemiology among US adults hospitalized for community-acquired pneumonia. *Vaccine* **2019**, *37*, 3352-3361, doi:10.1016/j.vaccine.2019.04.087.
44. Izquierdo, C.; Ciruela, P.; Hernández, S.; García-García, J.J.; Esteva, C.; Moraga-Llop, F.; Díaz-Conradi, A.; Martínez-Osorio, J.; Solé-Ribalta, A.; de Sevilla, M.F.; et al. Pneumococcal serotypes in children, clinical presentation and antimicrobial susceptibility in the PCV13 era. *Epidemiol. Infect.* **2020**, *148*, e279, doi:10.1017/s0950268820002708.
45. Jayaraman, R.; Varghese, R.; Kumar, J.L.; Neeravi, A.; Shanmugasundaram, D.; Ralph, R.; Thomas, K.; Veeraraghavan, B. Invasive pneumococcal disease in Indian adults: 11 years' experience. *J. Microbiol. Immunol. Infect.* **2019**, *52*, 736-742, doi:10.1016/j.jmii.2018.03.004.
46. Jayasinghe, S.; Menzies, R.; Chiu, C.; Toms, C.; Blyth, C.C.; Krause, V.; McIntyre, P. Long-term impact of a "3 + 0" schedule for 7- and 13-valent pneumococcal conjugate vaccines on invasive pneumococcal disease in Australia, 2002-2014. *Clin. Infect. Dis.* **2017**, *64*, 175-183, doi:10.1093/cid/ciw720.
47. Kaplan, S.L.; Barson, W.J.; Lin, P.L.; Romero, J.R.; Bradley, J.S.; Tan, T.Q.; Hoffman, J.A.; Givner, L.B.; Mason, E.O., Jr. Early trends for invasive pneumococcal infections in children after the introduction of the 13-valent pneumococcal conjugate vaccine. *Pediatr. Infect. Dis. J.* **2013**, *32*, 203-207, doi:10.1097/INF.0b013e318275614b.
48. Kaplan, S.L.; Barson, W.J.; Lin, P.L.; Romero, J.R.; Bradley, J.S.; Tan, T.Q.; Pannaraj, P.S.; Givner, L.B.; Hulten, K.G. Invasive Pneumococcal Disease in Children's Hospitals: 2014-2017. *Pediatrics* **2019**, *144*, e20190567, doi:10.1542/peds.2019-0567.
49. Kawaguchiya, M.; Urushibara, N.; Aung, M.S.; Morimoto, S.; Ito, M.; Kudo, K.; Sumi, A.; Kobayashi, N. Emerging non-PCV13 serotypes of noninvasive Streptococcus pneumoniae with macrolide resistance genes in northern Japan. *New Microbes New Infect* **2016**, *9*, 66-72, doi:10.1016/j.nmni.2015.11.001.
50. Kawaguchiya, M.; Urushibara, N.; Aung, M.S.; Morimoto, S.; Ito, M.; Kudo, K.; Kobayashi, N. Genetic diversity of pneumococcal surface protein A (PspA) in paediatric isolates of non-conjugate vaccine serotypes in Japan. *J. Med. Microbiol.* **2018**, *67*, 1130-1138, doi:10.1099/jmm.0.000775.
51. Kawaguchiya, M.; Urushibara, N.; Aung, M.S.; Shinagawa, M.; Takahashi, S.; Kobayashi, N. Serotype distribution, antimicrobial resistance and prevalence of pilus islets in pneumococci following the use of conjugate vaccines. *J. Med. Microbiol.* **2017**, *66*, 643-650, doi:10.1099/jmm.0.000479.
52. Kendall, B.A.; Dascomb, K.K.; Mehta, R.R.; Stockmann, C.; Mason, E.O.; Ampofo, K.; Pavia, A.T.; Byington, C.L. Early Streptococcus pneumoniae serotype changes in Utah adults after the introduction of PCV13 in children. *Vaccine* **2016**, *34*, 474-478, doi:10.1016/j.vaccine.2015.12.010.
53. Ktari, S.; Jmal, I.; Mroua, M.; Maalej, S.; Ben Ayed, N.E.; Mnif, B.; Rhimi, F.; Hammami, A. Serotype distribution and antibiotic susceptibility of *Streptococcus pneumoniae* strains in the south of Tunisia: a five-year study (2012-2016) of pediatric and adult populations. *Int. J. Infect. Dis.* **2017**, *65*, 110-115, doi:10.1016/j.ijid.2017.10.015.

54. Ladhani, S.N.; Collins, S.; Djennad, A.; Sheppard, C.L.; Borrow, R.; Fry, N.K.; Andrews, N.J.; Miller, E.; Ramsay, M.E. Rapid increase in non-vaccine serotypes causing invasive pneumococcal disease in England and Wales, 2000–17: a prospective national observational cohort study. *Lancet Infect. Dis.* **2018**, *18*, 441-451, doi:10.1016/S1473-3099(18)30052-5.
55. Latasa Zamalloa, P.; Sanz Moreno, J.C.; Ordobas Gavin, M.; Barranco Ordonez, M.D.; Insua Marisquerena, E.; Gil de Miguel, A.; Fernandez Chavez, A.C.; Garcia-Comas, L. Trends of invasive pneumococcal disease and its serotypes in the Autonomous Community of Madrid. *Enferm Infecc Microbiol Clin (Engl Ed)* **2018**, *36*, 612-620, doi:10.1016/j.eimc.2017.10.026.
56. Lawrence, J.; Gwee, A.; Quinlan, C. Pneumococcal haemolytic uraemic syndrome in the postvaccine era. *Arch. Dis. Child.* **2018**, *103*, 957-961, doi:10.1136/archdischild-2017-313923.
57. Leach, A.J.; Wigger, C.; Beissbarth, J.; Woltring, D.; Andrews, R.; Chatfield, M.D.; Smith-Vaughan, H.; Morris, P.S. General health, otitis media, nasopharyngeal carriage and middle ear microbiology in Northern Territory Aboriginal children vaccinated during consecutive periods of 10-valent or 13-valent pneumococcal conjugate vaccines. *Int. J. Pediatr. Otorhinolaryngol.* **2016**, *86*, 224-232.
58. LeBlanc, J.J.; ElSherif, M.; Ye, L.; MacKinnon-Cameron, D.; Ambrose, A.; Hatchette, T.F.; Lang, A.L.S.; Gillis, H.D.; Martin, I.; Demczuk, W.; et al. Streptococcus pneumoniae serotype 3 is masking PCV13-mediated herd immunity in Canadian adults hospitalized with community acquired pneumonia: A study from the Serious Outcomes Surveillance (SOS) Network of the Canadian immunization research Network (CIRN). *Vaccine* **2019**, *37*, 5466-5473, doi:10.1016/j.vaccine.2019.05.003.
59. LeBlanc, J.; El Sherif, M.; Ye, L.; MacKinnon-Cameron, D.; Ambrose, A.; Hatchette, T.F.; Lang, A.L.; Gillis, H.D.; Martin, I.; Demczuk, W.H.; et al. Age-stratified burden of pneumococcal community acquired pneumonia in hospitalised Canadian adults from 2010 to 2015. *BMJ Open Respir Res* **2020**, *7*, e000550, doi:10.1136/bmjresp-2019-000550.
60. Leibovitz, E.; David, N.; Ribitzky-Eisner, H.; Abo Madegam, M.; Abuabed, S.; Chodick, G.; Maimon, M.; Fruchtman, Y. The Epidemiologic, Microbiologic and Clinical Picture of Bacteremia among Febrile Infants and Young Children Managed as Outpatients at the Emergency Room, before and after Initiation of the Routine Anti-Pneumococcal Immunization. *Int. J. Environ. Res. Public Health* **2016**, *13*, 723, doi:10.3390/ijerph13070723.
61. LeMeur, J.B.; Lefebvre, B.; Proulx, J.F.; De Wals, P. Limited impact of pneumococcal vaccines on invasive pneumococcal disease in Nunavik (Quebec). *Can. J. Public Health.* **2019**, *110*, 36-43, doi:10.17269/s41997-018-0138-2.
62. Lepoutre, A.; Varon, E.; Georges, S.; Dorleans, F.; Janoir, C.; Gutmann, L.; Levy-Bruhl, D.; Microbiologists of Epibac; ORP Networks. Impact of the pneumococcal conjugate vaccines on invasive pneumococcal disease in France, 2001-2012. *Vaccine* **2015**, *33*, 359-366, doi:10.1016/j.vaccine.2014.11.011.
63. Levy, C.; Varon, E.; Picard, C.; Bechet, S.; Martinot, A.; Bonacorsi, S.; Cohen, R. Trends of pneumococcal meningitis in children after introduction of the 13-valent pneumococcal conjugate Vaccine in France. *Pediatr. Infect. Dis. J.* **2014**, *33*, 1216-1221, doi:10.1097/INF.0000000000000451.
64. Levy, C.; Varon, E.; Ouldali, N.; Wollner, A.; Thollot, F.; Corrard, F.; Werner, A.; Bechet, S.; Bonacorsi, S.; Cohen, R. Bacterial causes of otitis media with spontaneous perforation of the tympanic membrane in the era of 13 valent pneumococcal conjugate vaccine. *PLoS One* **2019**, *14*, e0211712, doi:10.1371/journal.pone.0211712.
65. Liese, J.G.; Schoen, C.; van der Linden, M.; Lehmann, L.; Goettler, D.; Keller, S.; Maier, A.; Segerer, F.; Rose, M.A.; Streng, A. Changes in the incidence and bacterial aetiology of paediatric parapneumonic pleural effusions/empyema in Germany, 2010-2017: a nationwide surveillance study. *Clin. Microbiol. Infect.* **2019**, *25*, 857-864, doi:10.1016/j.cmi.2018.10.020.
66. Lindstrand, A.; Galanis, I.; Darenberg, J.; Morfeldt, E.; Naucler, P.; Blennow, M.; Alfven, T.; Henriques-Normark, B.; Ortqvist, A. Unaltered pneumococcal carriage prevalence due to expansion of non-vaccine types of low invasive potential 8years after vaccine introduction in Stockholm, Sweden. *Vaccine* **2016**, *34*, 4565-4571.

67. Lo, S.W.; Gladstone, R.A.; van Tonder, A.J.; Lees, J.A.; du Plessis, M.; Benisty, R.; Givon-Lavi, N.; Hawkins, P.A.; Cornick, J.E.; Kwambana-Adams, B.; et al. Pneumococcal lineages associated with serotype replacement and antibiotic resistance in childhood invasive pneumococcal disease in the post-PCV13 era: an international whole-genome sequencing study. *Lancet Infect. Dis.* **2019**, *19*, 759-769.
68. Makwana, A.; Sheppard, C.; Borrow, R.; Fry, N.; Andrews, N.J.; Ladhani, S.N. Characteristics of Children With Invasive Pneumococcal Disease After the Introduction of the 13-valent Pneumococcal Conjugate Vaccine in England and Wales, 2010-2016. *Pediatr. Infect. Dis. J.* **2018**, *37*, 697-703, doi:10.1097/INF.0000000000001845.
69. Malaker, R.; Saha, S.; Hanif, M.; Ahmed, A.; Saha, S.; Hasanuzzaman, M.; Khondakar, T.; Islam, M.; Baqui, A.H.; Santosham, M.; et al. Invasive pneumococcal infections in children with nephrotic syndrome in Bangladesh. *Pediatr. Infect. Dis. J.* **2019**, *38*, 798-803, doi:10.1097/inf.0000000000002386.
70. Marcus, J.L.; Baxter, R.; Leyden, W.A.; Muthulingam, D.; Yee, A.; Horberg, M.A.; Klein, D.B.; Towner, W.J.; Chao, C.R.; Quesenberry, C.P., Jr.; et al. Invasive Pneumococcal Disease Among HIV-Infected and HIV-Uninfected Adults in a Large Integrated Healthcare System. *AIDS Patient Care STDS* **2016**, *30*, 463-470, doi:10.1089/apc.2016.0165.
71. Marrie, T.J.; Tyrrell, G.J.; Majumdar, S.R.; Eurich, D.T. Asplenic patients and invasive pneumococcal disease-how bad is it these days? *Int. J. Infect. Dis.* **2016**, *51*, 27-30, doi:10.1016/j.ijid.2016.08.022.
72. Massora, S.; Lessa, F.C.; Moiane, B.; Pimenta, F.C.; Mucavele, H.; Chauque, A.; Cossa, A.; Verani, J.R.; Tembe, N.; da Gloria Carvalho, M.; et al. Invasive disease potential of Streptococcus pneumoniae serotypes before and after 10-valent pneumococcal conjugate vaccine introduction in a rural area, southern Mozambique. *Vaccine* **2019**, *37*, 7470-7477, doi:10.1016/j.vaccine.2019.09.079.
73. Mendes, R.E.; Hollingsworth, R.C.; Costello, A.; Jones, R.N.; Isturiz, R.E.; Hewlett, D., Jr.; Farrell, D.J. Noninvasive *Streptococcus pneumoniae* serotypes recovered from hospitalized adult patients in the United States (2009-2012). *Antimicrob. Agents Chemother.* **2015**, *59*, 5595-5601.
74. Méndez-Lage, S.; Losada-Castillo, I.; Agulla-Budiño, A. [Streptococcus pneumoniae: serotype distribution, antimicrobial susceptibility, risk factors and mortality in Galicia over a two year-period]. *Enferm. Infecc. Microbiol. Clin.* **2015**, *33*, 579-584, doi:10.1016/j.eimc.2015.01.010.
75. Menendez, R.; Espana, P.P.; Perez-Trallero, E.; Uranga, A.; Mendez, R.; Cilloniz, C.; Marimon, J.M.; Cifuentes, I.; Mendez, C.; Torres, A. The burden of PCV13 serotypes in hospitalized pneumococcal pneumonia in Spain using a novel urinary antigen detection test. CAPA study. *Vaccine* **2017**, *35*, 5264-5270, doi:10.1016/j.vaccine.2017.08.007.
76. Mokaddas, E.; Albert, M.J. Impact of pneumococcal conjugate vaccines on burden of invasive pneumococcal disease and serotype distribution of *Streptococcus pneumoniae* isolates: an overview from Kuwait. *Vaccine* **2012**, *30 suppl 6*, G37-40, doi:10.1016/j.vaccine.2012.10.061.
77. Mokaddas, E.; Albert, M.J. Serotype distribution and penicillin-non-susceptibility of *Streptococcus pneumoniae* causing invasive diseases in Kuwait: a 10-year study of impact of pneumococcal conjugate vaccines. *Expert Rev Vaccines* **2016**, *15*, 1337-1345, doi:10.1080/14760584.2016.1198698.
78. Moore, C.E.; Paul, J.; Foster, D.; Mahar, S.A.; Griffiths, D.; Knox, K.; Peto, T.E.; Walker, A.S.; Crook, D.W.; Oxford Invasive Pneumococcal Surveillance, G. Reduction of invasive pneumococcal disease 3 years after the introduction of the 13-valent conjugate vaccine in the Oxfordshire region of England. *J. Infect. Dis.* **2014**, *210*, 1001-1011.
79. Moore, M.R.; Link-Gelles, R.; Schaffner, W.; Lynfield, R.; Lexau, C.; Bennett, N.M.; Petit, S.; Zansky, S.M.; Harrison, L.H.; Reingold, A. Effect of use of 13-valent pneumococcal conjugate vaccine in children on invasive pneumococcal disease in children and adults in the USA: analysis of multisite, population-based surveillance. *Lancet Infect. Dis.* **2015**, *15*, 301-309, doi:10.1016/S1473-3099(14)71081-3.

80. Nakano, S.; Fujisawa, T.; Ito, Y.; Chang, B.; Suga, S.; Noguchi, T.; Yamamoto, M.; Matsumura, Y.; Nagao, M.; Takakura, S. Serotypes, antimicrobial susceptibility, and molecular epidemiology of invasive and non-invasive Streptococcus pneumoniae isolates in paediatric patients after the introduction of 13-valent conjugate vaccine in a nationwide surveillance study conducted in Japan in 2012–2014. *Vaccine* **2016**, *34*, 67-76, doi:10.1016/j.vaccine.2015.11.015.
81. Naziat, H.; Saha, S.; Islam, M.; Saha, S.; Uddin, M.J.; Hussain, M.; Luby, S.P.; Darmstadt, G.L.; Whitney, C.G.; Gessner, B.D.; et al. Epidemiology of otitis media with otorrhea among Bangladeshi children: baseline study for future assessment of pneumococcal conjugate vaccine impact. *Pediatr. Infect. Dis. J.* **2018**, *37*, 715-721, doi:10.1097/INF.0000000000002077.
82. Negash, A.A.; Asrat, D.; Abebe, W.; Hailemariam, T.; Gebre, M.; Aseffa, A.; Vaneechoutte, M. Pneumococcal serotype 19A is the major cause of pediatric acute otitis media with ruptured tympanic membrane in Addis Ababa, Ethiopia, 5 years after the introduction of the ten-valent pneumococcal conjugate vaccine. *Int. J. Pediatr. Otorhinolaryngol.* **2019**, *126*, 109638, doi:10.1016/j.ijporl.2019.109638.
83. Noguchi, S.; Yatera, K.; Akata, K.; Chang, B.; Ikegami, H.; Hata, R.; Yamasaki, K.; Kawanami, T.; Mukae, H. Distribution and annual changes in the proportion of Streptococcus pneumoniae serotypes in Japanese adults with pneumococcal pneumonia from 2011 to 2017. *J Infect Chemother* **2019**, *25*, 925-929, doi:10.1016/j.jiac.2019.07.007.
84. Ochoa-Gondar, O.; Figuerola-Massana, E.; Vila-Corcoles, A.; Aguirre, C.A.; de Diego, C.; Satue, E.; Gomez, F.; Raga, X.; Group, E.S. Epidemiology of *Streptococcus pneumoniae* causing acute otitis media among children in Southern Catalonia throughout 2007-2013: incidence, serotype distribution and vaccine's effectiveness. *Int. J. Pediatr. Otorhinolaryngol.* **2015**, *79*, 2104-2108, doi:10.1016/j.ijporl.2015.09.022.
85. Olarte, L.; Ampofo, K.; Stockmann, C.; Mason, E.O.; Daly, J.A.; Pavia, A.T.; Byington, C.L. Invasive pneumococcal disease in infants younger than 90 days before and after introduction of PCV7. *Pediatrics* **2013**, *132*, e17-24.
86. Olarte, L.; Barson, W.J.; Barson, R.M.; Romero, J.R.; Bradley, J.S.; Tan, T.Q.; Givner, L.B.; Hoffman, J.A.; Lin, P.L.; Hulten, K.G.; et al. Pneumococcal Pneumonia Requiring Hospitalization in US Children in the 13-Valent Pneumococcal Conjugate Vaccine Era. *Clin. Infect. Dis.* **2017**, *64*, 1699-1704, doi:10.1093/cid/cix115.
87. Olarte, L.; Barson, W.J.; Bradley, J.S.; Tan, T.Q.; Lin, P.L.; Romero, J.R.; Givner, L.B.; Hoffman, J.A.; Hulten, K.G.; Mason, E.O.; et al. Invasive Pneumococcal Disease in Infants Aged 0-60 Days in the United States in the 13-Valent Pneumococcal Conjugate Vaccine Era. *J Pediatric Infect Dis Soc* **2018**, *7*, 249-252, doi:10.1093/jpids/pix034.
88. Oligbu, G.; Collins, S.; Djennad, A.; Sheppard, C.L.; Fry, N.K.; Andrews, N.J.; Borrow, R.; Ramsay, M.E.; Ladhani, S.N. Effect of Pneumococcal Conjugate Vaccines on Pneumococcal Meningitis, England and Wales, July 1, 2000-June 30, 2016. *Emerg. Infect. Dis.* **2019**, *25*, 1708-1718, doi:10.3201/eid2509.180747.
89. Ouldali, N.; Varon, E.; Levy, C.; Angoulvant, F.; Georges, S.; Ploy, M.C.; Kempf, M.; Cremniter, J.; Cohen, R.; Bruhl, D.L.; et al. Invasive pneumococcal disease incidence in children and adults in France during the pneumococcal conjugate vaccine era: an interrupted time-series analysis of data from a 17-year national prospective surveillance study. *Lancet Infect. Dis.* **2021**, *21*, 137-147, doi:10.1016/S1473-3099(20)30165-1.
90. Pick, H.; Daniel, P.; Rodrigo, C.; Bewick, T.; Ashton, D.; Lawrence, H.; Baskaran, V.; Edwards-Pritchard, R.C.; Sheppard, C.; Eletu, S.D.; et al. Pneumococcal serotype trends, surveillance and risk factors in UK adult pneumonia, 2013-18. *Thorax* **2020**, *75*, 38-49, doi:10.1136/thoraxjnl-2019-213725.
91. Pinto, T.C.A.; Neves, F.P.G.; Souza, A.R.V.; Oliveira, L.M.A.; Costa, N.S.; Castro, L.F.S.; Mendonca-Souza, C.R.V.; Peralta, J.M.; Teixeira, L.M. Evolution of Penicillin Non-susceptibility Among Streptococcus pneumoniae Isolates Recovered From Asymptomatic Carriage and Invasive Disease Over 25 years in Brazil, 1990-2014. *Front. Microbiol.* **2019**, *10*, 486, doi:10.3389/fmicb.2019.00486.

92. Prato, R.; Fortunato, F.; Cappelli, M.G.; Chironna, M.; Martinelli, D. Effectiveness of the 13-valent pneumococcal conjugate vaccine against adult pneumonia in Italy: a case-control study in a 2-year prospective cohort. *BMJ Open* **2018**, *8*, e019034, doi:10.1136/bmjopen-2017-019034.
93. Richter, S.S.; Heilmann, K.P.; Dohrn, C.L.; Riahi, F.; Diekema, D.J.; Doern, G.V. Pneumococcal serotypes before and after introduction of conjugate vaccines, United States, 1999-2011(1.). *Emerg. Infect. Dis.* **2013**, *19*, 1074-1083, doi:10.3201/eid1907.121830 [doi].
94. Richter, L.; Schmid, D.; Kanitz, E.E.; Zwazl, I.; Pollabauer, E.; Jasinska, J.; Burgmann, H.; Kundi, M.; Wiedermann, U. Invasive pneumococcal diseases in children and adults before and after introduction of the 10-valent pneumococcal conjugate vaccine into the Austrian national immunization program. *PLoS One* **2019**, *14*, e0210081, doi:10.1371/journal.pone.0210081.
95. Rudnick, W.; Liu, Z.; Shigayeva, A.; Low, D.E.; Green, K.; Plevneshi, A.; Devlin, R.; Downey, J.; Katz, K.; Kitai, I.; et al. Pneumococcal vaccination programs and the burden of invasive pneumococcal disease in Ontario, Canada, 1995-2011. *Vaccine* **2013**, *31*, 5863-5871.
96. Sanz-Herrero, F.; Gimeno-Cardona, C.; Tormo-Palop, N.; Fernandez-Fabrellas, E.; Briones, M.L.; Cervera-Juan, A.; Blanquer-Olivas, J. The potential role of 13-valent pneumococcal conjugate vaccine in preventing respiratory complications in bacteraemic pneumococcal community-acquired pneumonia. *Vaccine* **2016**, *34*, 1847-1852, doi:10.1016/j.vaccine.2016.01.038.
97. Sanz, J.C.; Rodríguez-Avial, I.; Ríos, E.; García-Comas, L.; Ordobás, M.; Cercenado, E. Increase of serotype 8, ST53 clone, as the prevalent strain of Streptococcus pneumoniae causing invasive disease in Madrid, Spain (2012-2015). *Enferm. Infecc. Microbiol. Clin.* **2020**, *38*, 105-110, doi:10.1016/j.eimc.2019.05.006.
98. Sheppard, C.; Fry, N.K.; Mushtaq, S.; Woodford, N.; Reynolds, R.; Janes, R.; Pike, R.; Hill, R.; Kimuli, M.; Staves, P.; et al. Rise of multidrug-resistant non-vaccine serotype 15A Streptococcus pneumoniae in the United Kingdom, 2001 to 2014. *Euro Surveill.* **2016**, *21*, 30423, doi:10.2807/1560-7917.ES.2016.21.50.30423.
99. Shoji, H.; Domenech, A.; Simonetti, A.F.; Gonzalez, A.; Garcia-Somoza, D.; Cubero, M.; Marti, S.; Maeda, M.; Tubau, F.; Linares, J.; et al. The Alere BinaxNOW Pneumococcal Urinary Antigen Test: Diagnostic Sensitivity for Adult Pneumococcal Pneumonia and Relationship to Specific Serotypes. *J. Clin. Microbiol.* **2018**, *56*, e00787-00717, doi:10.1128/JCM.00787-17.
100. Silva-Costa, C.; Brito, M.J.; Pinho, M.D.; Friaes, A.; Aguiar, S.I.; Ramirez, M.; Melo-Cristino, J. Pediatric complicated pneumonia caused by *Streptococcus pneumoniae* serotype 3 in 13-valent pneumococcal conjugate vaccinees, Portugal, 2010-2015. *Emerg. Infect. Dis.* **2018**, *24*, 1307-1314, doi:10.3201/eid2407.180029.
101. Silva-Costa, C.; Brito, M.J.; Aguiar, S.I.; Lopes, J.P.; Ramirez, M.; Melo-Cristino, J.; Portuguese Group for the Study of Streptococcal, I.; Portuguese Study Group of Invasive Pneumococcal Disease of the Pediatric Infectious Disease, S. Dominance of vaccine serotypes in pediatric invasive pneumococcal infections in Portugal (2012-2015). *Sci. Rep.* **2019**, *9*, 6, doi:10.1038/s41598-018-36799-x.
102. Slotved, H.C.; Dalby, T.; Hoffmann, S. Invasive pneumococcal isolates from Danish infants (0 - 90 Days) during the years 1943 to 2013. *PLoS One* **2014**, *9*, e106180, doi:10.1371/journal.pone.0106180.
103. Slotved, H.C.; Dalby, T.; Hoffmann, S. The effect of pneumococcal conjugate vaccines on the incidence of invasive pneumococcal disease caused by ten non-vaccine serotypes in Denmark. *Vaccine* **2016**, *34*, 769-774, doi:10.1016/j.vaccine.2015.12.056.
104. Steens, A.; Bergsaker, M.A.; Aaberge, I.S.; Ronning, K.; Vestrheim, D.F. Prompt effect of replacing the 7-valent pneumococcal conjugate vaccine with the 13-valent vaccine on the epidemiology of invasive pneumococcal disease in Norway. *Vaccine* **2013**, *31*, 6232-6238, doi:S0264-410X(13)01408-4 pii10.1016/j.vaccine.2013.10.032 doi.
105. Su, L.H.; Kuo, A.J.; Chia, J.H.; Li, H.C.; Wu, T.L.; Feng, Y.; Chiu, C.H. Evolving pneumococcal serotypes and sequence types in relation to high antibiotic stress and conditional pneumococcal immunization. *Sci. Rep.* **2015**, *5*, 15843, doi:10.1038/srep15843.

106. Su, W.J.; Lo, H.Y.; Chang, C.H.; Chang, L.Y.; Chiu, C.H.; Lee, P.I.; Lu, C.Y.; Hsieh, Y.C.; Lai, M.S.; Lin, T.Y. Effectiveness of Pneumococcal Conjugate Vaccines of Different Valences Against Invasive Pneumococcal Disease Among Children in Taiwan: A Nationwide Study. *Pediatr. Infect. Dis. J.* **2016**, *35*, e124-133, doi:10.1097/INF.0000000000001054.
107. Suaya, J.A.; Mendes, R.E.; Sings, H.L.; Arguedas, A.; Reinert, R.R.; Jodar, L.; Isturiz, R.E.; Gessner, B.D. Streptococcus pneumoniae serotype distribution and antimicrobial nonsusceptibility trends among adults with pneumonia in the United States, 20092017. *J. Infect.* **2020**, *81*, 557-566, doi:10.1016/j.jinf.2020.07.035.
108. Takeuchi, N.; Naito, S.; Ohkusu, M.; Abe, K.; Shizuno, K.; Takahashi, Y.; Omata, Y.; Nakazawa, T.; Takeshita, K.; Hishiki, H.; et al. Epidemiology of hospitalised paediatric community-acquired pneumonia and bacterial pneumonia following the introduction of 13-valent pneumococcal conjugate vaccine in the national immunisation programme in Japan. *Epidemiol. Infect.* **2020**, *148*, e91, doi:10.1017/s0950268820000813.
109. Turner, P.; Leab, P.; Ly, S.; Sao, S.; Miliya, T.; Heffelfinger, J.D.; Batmunkh, N.; Lessa, F.C.; Walldorf, J.A.; Hyde, T.B.; et al. Impact of 13-valent pneumococcal conjugate vaccine on colonization and invasive disease in Cambodian children. *Clin. Infect. Dis.* **2020**, *70*, 1580-1588, doi:10.1093/cid/ciz481.
110. Ubukata, K.; Takata, M.; Morozumi, M.; Chiba, N.; Wajima, T.; Hanada, S.; Shouji, M.; Sakuma, M.; Iwata, S. Effects of pneumococcal conjugate vaccine on genotypic penicillin resistance and serotype changes, Japan, 2010-2017. *Emerg. Infect. Dis.* **2018**, *24*, 2010-2020, doi:10.3201/eid2411.180326.
111. Ubukata, K.; Morozumi, M.; Sakuma, M.; Takata, M.; Mokuno, E.; Tajima, T.; Iwata, S.; Group, A.O.M.S.S. Etiology of Acute Otitis Media and Characterization of Pneumococcal Isolates After Introduction of 13-Valent Pneumococcal Conjugate Vaccine in Japanese Children. *Pediatr. Infect. Dis. J.* **2018**, *37*, 598-604, doi:10.1097/INF.0000000000001956.
112. Uddén, F.; Rünow, E.; Slotved, H.C.; Fuursted, K.; Ahl, J.; Riesbeck, K. Characterization of Streptococcus pneumoniae detected in clinical respiratory tract samples in southern Sweden 2 to 4 years after introduction of PCV13. *J. Infect.* **2021**, *83*, 190-196, doi:10.1016/j.jinf.2021.05.031.
113. van der Linden, M.; Imohl, M.; Perniciaro, S. Limited indirect effects of an infant pneumococcal vaccination program in an aging population. *PLoS One* **2019**, *14*, e0220453, doi:10.1371/journal.pone.0220453.
114. van der Linden, M.; Falkenhorst, G.; Perniciaro, S.; Imohl, M. Effects of Infant Pneumococcal Conjugate Vaccination on Serotype Distribution in Invasive Pneumococcal Disease among Children and Adults in Germany. *PLoS One* **2015**, *10*, e0131494, doi:10.1371/journal.pone.0131494.
115. van Hoek, A.J.; Andrews, N.; Waight, P.A.; George, R.; Miller, E. Effect of serotype on focus and mortality of invasive pneumococcal disease: coverage of different vaccines and insight into non-vaccine serotypes. *PLoS One* **2012**, *7*, e39150, doi:10.1371/journal.pone.0039150.
116. Vila Córcoles, Á.; Dacosta Moreira, C.; de Diego Cabanes, C.; Ochoa Gondar, O.; Raga Gutiérrez, M.; Gómez Bertomeu, F.; Raga Luria, X.; Figuerola Massana, E. [Epidemiology of invasive pneumococcal disease in Tarragona, Spain, 2012-2015: incidence, lethality and serotype-coverage for distinct antipneumococcal vaccine formulations]. *Rev. Esp. Salud Publica* **2018**, *92*, e201810073.
117. von Gottberg, A.; de Gouveia, L.; Tempia, S.; Quan, V.; Meiring, S.; von Mollendorf, C.; Madhi, S.A.; Zell, E.R.; Verani, J.R.; O'Brien, K.L.; et al. Effects of vaccination on invasive pneumococcal disease in South Africa. *N. Engl. J. Med.* **2014**, *371*, 1889-1899, doi:10.1056/NEJMoa1401914.
118. Wagenvoort, G.H.; Sanders, E.A.; Vlaminckx, B.J.; Elberse, K.E.; de Melker, H.E.; van der Ende, A.; Knol, M.J. Invasive pneumococcal disease: Clinical outcomes and patient characteristics 2-6 years after introduction of 7-valent pneumococcal conjugate vaccine compared to the pre-vaccine period, the Netherlands. *Vaccine* **2016**, *34*, 1077-1085, doi:10.1016/j.vaccine.2015.12.066.
119. Wagenvoort, G.H.; Knol, M.J.; de Melker, H.E.; Vlaminckx, B.J.; van der Ende, A.; Rozenbaum, M.H.; Sanders, E.A. Risk and outcomes of invasive pneumococcal disease in

adults with underlying conditions in the post-PCV7 era, The Netherlands. *Vaccine* **2016**, *34*, 334-340, doi:10.1016/j.vaccine.2015.11.048.

120. Waight, P.A.; Andrews, N.J.; Ladhani, S.N.; Sheppard, C.L.; Slack, M.P.; Miller, E. Effect of the 13-valent pneumococcal conjugate vaccine on invasive pneumococcal disease in England and Wales 4 years after its introduction: an observational cohort study. *Lancet Infect. Dis.* **2015**, *15*, 535-543.
121. Wang, J.; Liu, F.; Ao, P.; Li, X.; Zheng, H.; Wu, D.; Zhang, N.; Yu, J.; Yuan, J.; Wu, X. Detection of Serotype Distribution and Drug Resistance of Streptococcus Pneumoniae Isolated From Pediatric Patients. *Lab. Med.* **2017**, *48*, 39-45, doi:10.1093/labmed/lmw059.
122. Weinberger, R.; Falkenhorst, G.; Bogdan, C.; van der Linden, M.; Imohl, M.; von Kries, R. Incidence of invasive pneumococcal disease in 5-15 year old children with and without comorbidities in Germany after the introduction of PCV13: Implications for vaccinating children with comorbidities. *Vaccine* **2015**, *33*, 6617-6621, doi:10.1016/j.vaccine.2015.10.102.
123. Weinberger, R.; von Kries, R.; van der Linden, M.; Rieck, T.; Siedler, A.; Falkenhorst, G. Invasive pneumococcal disease in children under 16 years of age: Incomplete rebound in incidence after the maximum effect of PCV13 in 2012/13 in Germany. *Vaccine* **2018**, *36*, 572-577, doi:10.1016/j.vaccine.2017.11.085.
124. Wijayasri, S.; Hillier, K.; Lim, G.H.; Harris, T.M.; Wilson, S.E.; Deeks, S.L. The shifting epidemiology and serotype distribution of invasive pneumococcal disease in Ontario, Canada, 2007-2017. *PLoS One* **2019**, *14*, e0226353, doi:10.1371/journal.pone.0226353.
125. Yun, K.W.; Choi, E.H.; Lee, H.J.; Kang, J.H.; Kim, K.H.; Kim, D.S.; Kim, Y.J.; Eun, B.W.; Oh, S.H.; Cho, H.K.; et al. Genetic structures of invasive Streptococcus pneumoniae isolates from Korean children obtained between 1995 and 2013. *BMC Infect. Dis.* **2018**, *18*, 268, doi:10.1186/s12879-018-3177-7.
126. Zintgraff, J.; Fossati, S.; Pereira, C.S.; Veliz, O.; Regueira, M.; Moscoloni, M.A.; Irazu, L.; Lara, C.; Napoli, D.; Argentina Spn Working, G. Distribution of PCV13 and PPSV23 *Streptococcus pneumoniae* serotypes in Argentinean adults with invasive disease, 2013-2017. *Rev. Argent. Microbiol.* **2020**, *52*, 189-194, doi:10.1016/j.ram.2019.11.004.
127. Ziv, O.; Kraus, M.; Holcberg, R.; Dinur, A.B.; Kordeluk, S.; Kaplan, D.; Rosenblatt, H.N.; Ben-Shimol, S.; Greenberg, D.; Leibovitz, E. Acute otitis media in infants younger than two months of age: Epidemiologic and microbiologic characteristics in the era of pneumococcal conjugate vaccines. *Int. J. Pediatr. Otorhinolaryngol.* **2019**, *119*, 123-130, doi:10.1016/j.ijporl.2019.01.031.
